# Community Profiling of Culturable Fluorescent Pseudomonads in the Rhizosphere of Green Gram (*Vigna radiata* L.)

**DOI:** 10.1371/journal.pone.0108378

**Published:** 2014-10-03

**Authors:** Rupak K. Sarma, Animesh Gogoi, Budheswar Dehury, Rajal Debnath, Tarun C. Bora, Ratul Saikia

**Affiliations:** 1 Biotechnology Division, CSIR-North East Institute of Science and Technology, Jorhat, Assam, India; 2 Department of Agricultural Biotechnology, Assam Agricultural University, Jorhat, Assam, India; Ghent University, Belgium

## Abstract

Study on microbial diversity in the unexplored rhizosphere is important to understand their community structure, biology and ecological interaction with the host plant. This research assessed the genetic and functional diversity of fluorescent pseudomonads [FP] in the green gram rhizophere. One hundred and twenty types of morphologically distinct fluorescent pseudomonads were isolated during vegetative as well as reproductive growth phase of green gram. Rep PCR, ARDRA and RISA revealed two distinct clusters in each case at 75, 61 and 70% similarity coefficient index respectively. *16S rRNA* partial sequencing analysis of 85 distantly related fluorescent pseudomonads depicted *Pseudomonas aeruginosa* as the dominant group. Out of 120 isolates, 23 (19%) showed antagonistic activity towards phytopathogenic fungi. These bacterial isolates showed varied production of salicylic acid, HCN and chitinase, 2, 4-diacetylphloroglucinol (DAPG), phenazine-1-carboxylic acid (PCA) and pyoluteorin (PLT). Production efficiency of inherent level of plant growth promoting (PGP) traits among the 120 isolates demonstrated that 10 (8%) solubilised inorganic phosphates, 25 (20%) produced indoles and 5 (4%) retained ACC deaminase activity. *Pseudomonas aeruginosa* GGRJ21 showed the highest production of all antagonistic and plant growth promoting (PGP) traits. In a greenhouse experiment, GGRJ21 suppressed root rot disease of green gram by 28–93% (p = 0.05). Consistent up regulation of three important stress responsive genes, i.e., *acdS*, *KatA* and *gbsA* and elevated production efficiency of different PGP traits could promote GGRJ21 as a potent plant growth regulator.

## Introduction

Fluorescent pseudomonads (FP) are one of the most diverse and ecologically significant groups under γ-proteobacteria that has been well studied in relation to their beneficial interactions with plants [1]. This ubiquitous bacterial group is widely accepted as most prominent plant growth promoting rhizobacteria (PGPR) [2], biocontrol agent [3] and a potential agent that may stimulate plant growth and development under varied abiotic stress conditions [4]–[6]. In the recent years, a wide attention was paid to decipher the diversity of fluorescent pseudomonads with keen reference to their biocontrol and biofertilizing abilities. Despite of other PGPR and non fluorescent pseudomonad isolates, wide recognition of fluorescent pseudomonads as potent plant growth promoter as well as biocontrol agent are mainly due to: 1) higher rhizosphere competence, i.e. extensive colonization in the ecto- and endorhizosphere when introduced through seed inoculation [7], [8] and 2) production efficiency of different secondary metabolites that can inhibit other microorganisms [1], [3]. Therefore, exploration of genetic and functional diversity of FPs from crop rhizosphere has great practical importance, with relevance to their application as effective biofertilizing and biocontrol agents.

The biocontrol activity of FP against different phytopathogens is mainly due to the production of diverse types of extracellular metabolites and antibiotic compounds [3]. Different phenazines, phenolics, polyketides, pyrrole-type compounds and siderophore from fluorescent pseudomonads render synergistic effect against the pathogenic microorganisms [9], [10]. Voisard et al. [11] and Keel et al. [12] reported the detrimental effect of fluorescent pseudomonads generated HCN and 2, 4-diacetylphloroglucinol (DAPG) against different soil borne phytopathogenic fungi. Similarly inherent production efficiency of indoles, 1-aminocyclopropane-1-carboxylate (ACC) deaminase, as well as phosphate solubilizing capability helps to place most of the γ-proteobacteria under PGPR class [4], [13], [14]. During the last decade several workers have reported induced drought tolerance in plants using *Pseudomonas* spp. [15]–[17]. Although the role of *Pseudomonas* spp. on water stress tolerance is not a new area to excavate; however to the best of our knowledge, very scanty amount of work is yet available on the amelioration of water stress through the use fluorescent pseudomonad isolates in acidic soils of North East India [4]. The genetic diversity and functional characterization of this large group in rhizosphere soils of different plants, *viz*., rice [18], [19], cotton [20], banana [13], wheat [21], and canola [14] have been already reported in different parts of the world. However, there is no report on total species richness of the ubiquitous group from rhizosphere of green gram. Green gram rhizosphere may sustain a rich repository of FP isolates that can be beneficial for plant health promotion. Previously we had reported the diversity of alkaline proteinase producing fluorescent pseudomonads from rhizosphere of green gram [22]. This was only a concise report on genetic diversity based on alkaline proteinase production. Thus, with continuation of the previous work, the present research was undertaken to investigate: (i) the total genetic diversity of green gram rhizosphere adhered fluorescent pseudomonads through PCR based molecular tools, *i.e.*, rep PCR (BOX-PCR and ERIC-PCR), amplified ribosomal DNA restriction analysis (ARDRA), ribosomal intergenic space analysis (RISA), and *16S rRNA* sequence analysis, (ii) functional diversity with relation to biocontrol and PGP traits along with their nature in water stress tolerance and (iii) mRNA expression level of three important drought responsive genes, *acdS*, *KatA* and *gbsA* in the stress tolerant isolate by real time quantitative polymerase chain reaction (qPCR).

## Materials and Methods

### Soil sampling and isolation of bacteria

Rhizosphere adhering soil samples were collected from ten different locations of green gram cultivating areas of Jorhat district of Assam, located in 26.75°N and 94.22°E of North East India. Sampling sites were selected based on minimal annual precipitation, *i.e* mainly drought prone areas. Sampling was carried out during the month of October (vegetative growth phase) and February (reproductive growth phase), 2011–2012. Soils were clay loam in texture with pH of 3.5 to 4. The soil samples from each location were combined and passed through 0.2 cm sieve and preserved at 4°C until use. A total of 120 fluorescent pseudomonad colonies were obtained upon growth on King’s B agar (KB) and *Pseudomonas* isolation agar (Hi Media, Mumbai, India) medium by incubating at 30±2°C for 24 hours The isolates were stored in 20% glycerol stock at −80°C until use.

### Ethics statement

Since the fields were public agricultural land; therefore, no further specific permission was required for obtaining samples from these locations.

### Microbial strains

Fungal pathogens *Fusarium oxysporum* f. sp. *raphani* (FoRN5), *Fusarium oxysporum* f. sp. *ciceri* (FocRs9), *Fusarium semitectum* (FsNJ9) and *Rhizoctonia solani* (RsNJ10) were obtained from the Culture Bank of Biotechnology Division, North-East Institute of Science Technology, Jorhat, Assam, India.

### Morphological and biochemical characterization

Isolates were gram stained and examined under light microscope. Biochemical characterization, *viz* fluorescent pigments, motility, nitrate reduction, catalase, oxidase, methyl red, starch hydrolysis, nitrate reduction and gelatin liquification tests were carried out with five replications as described in Bergey’s manual of determinative bacteriology [23].

### Genotypic Analysis

Genomic DNA of 120 fluorescent pseudomonads were extracted by GenElute Bacterial Genomic DNA Extraction kit (Sigma, USA), following the Manufacturer’s protocol. The DNA purity and quantity were checked by spectrophotometer at 260 and 280 nm. The genotypic analysis of 120 *Pseudomonas* strains were carried out by rep PCR using BOX-AIR1 primer (5′CTACGGCAAGGCGACGCTGACG3′) as described by Louws et al. 1994 [24] as well as ERIC F (5′AAGTAAGTGACTGGGGTGAGCG3′) and ERIC R (5′TGTAAGCTCCTGGGGATTCAĆ) as mentioned earlier by de Bruijn, 1992 [25] with three repetitions. A 10 µl PCR product together with 500 bp DNA marker (Bangalore Genei, Banglore, India) was separated on a 1.5% agarose gel stained with ethidium bromide in 1x TAE. A snapshot of the gel was taken by gel documentation system (UVP BioImaging system, Upland, California, USA) and stored as TIFF file for further analysis.

### Amplified ribosomal DNA restriction analysis (ARDRA)

Amplification of *16S rRNA* region was performed by using bacterial universal primers P^A^ (5′AGAGTTTGATCCTGGCTAG3′) and rP2 (5′ACGGCTACCTTGTTACGACTT3′) as described earlier by Edwards et al. 1989 [26]. Twenty microliters (100 ng) of purified *16S rRNA* PCR products were digested for 2 h with 1.5 U of *Hae*III, *Alu*I and *Msp*I restriction endonucleases respectively, as recommended by the manufacturer (Banglore Genei, Banglore, India). The restriction fragments were analyzed on a 2.5% agarose gel in 1X TAE electrophoresis buffer containing 10 µg ml^−1^ ethidium bromide and run at 40 V for 3 h. Whole experiment was repeated thrice to avoid any experimental error.

### Ribosomal intergenic space analysis (RISA)

The 16S–23S rDNA intergenic spacer region was amplified with universal primers G1 (5′GAAGTCGTAACAAGG3′) and L1 (5′CAAGGCATCCACCGT3′), as reported by Jensen et al. 1993 [27] with three repetitions. Purified amplicons (100 ng) were digested with single tetra cutter, *Msp*I. The products were separated on a (1.5%) agarose gel and run for 3 h at 40 V. The Gel was documented and stored as TIFF file for further analysis.

### Molecular Phylogenetic Analysis

Out of 120 bacterial isolates, 85 distantly related isolates were selected on the basis of clustering by rep PCR, RISA and ARDRA analysis. The clustering methods are described later. Furthermore, PCR amplified *16S rRNA* gene from the bacterial isolates was purified using Wizard PCR Preps (Promega, Madison, WI, USA) and then sequenced with an Applied Biosystems 310 automatic sequencer (Foster City, CA, USA). The ABI Prism dye terminator sequencing kits were used with the same primers used in *16S rRNA* amplification. Edited sequences were submitted in NCBI GenBank and accession numbers were obtained for the same. The reference *16S rRNA* gene sequences (*P. aeruginosa* NR 026078, *P. otitidis* NR 043289, *P. fulva* NR 040859, *P. monteilii* NR 024910, *P geniculata* NR024708) of NCBI GenBank along with our own sequenced bacterial isolates were subjected for phylogenetic inference.

### *In* *vitro* Screening for Antimicrobial Activity

Fluorescent pseudomonad isolates were tested for *in vitro* antagonism towards phyto pathogenic fungi, i.e. *F. oxysporum* f. sp. *raphani*, *F. oxysporum* f. sp. *ciceri*, *F. semitectum* and *R. solani* through agar well diffusion assay on potato dextrose agar [28] with three replicates for each bacterium. The minimum inhibitory concentration (MIC), *i.e.* the lowest concentration of the bacterial secondary metabolite was determined through batch cultures containing different volumes of 48 h old crude bacterial supernatant (1×10^9^ cfu ml^−1^) against all the phytopathogens (1×10^6^ conidia ml^−1^). Thus 60 µl was calculated to be the average MIC and loaded on to wells (5 mm diameter) of PDA plates pre inoculated with fungal spore suspension (1×10^6^ conidia ml^−1^). Assay plates were incubated at 28°C for 3 days and inhibition zone were recorded.

### Screening for Antimicrobial Traits

#### Assay for HCN

Quantitative assay for HCN production was carried out as described earlier by Kremer and Souissi, 2001 [29] with slight modification. Whatman No. 1 filter paper strips of 9 cm long and 0.5 cm wide, pre-equilibrated with alkaline picrate solution (0.5% picric acid in 2% sodium carbonate) were placed inside the conical flasks with bacterial culture in overhang position and incubated in a rotary shaker at 30°C for 48 h. Filter papers with color change from orange to yellow were removed, extracted by 5 ml 1.0 M NaOH and titrated with 4.25 ml acetic acid. Extracted cyanide into NaOH was further allowed to react with barbituric acid - pyridine reagent and absorbance was read at 575 nm. HCN was quantified as nmoles mg^−1^ cellular protein. The experiment was repeated thrice for each bacterium.

#### Chitinase Assay

The fluorescent pseudomonad isolates were grown in 100 ml of colloidal chitin supplemented peptone medium (colloidal chitin 0.2%, glucose 0.5%, peptone 0.2%, K_2_HPO_4_ 0.1%, MgSO_4_7H_2_O 0.05% and NaCl 0.05%, pH 6.8) at 28±1°C. Bacterial cultures were centrifuged at 12000×g for 15 minutes. Optical density of the supernatants was read at 280 nm (Specord 200, Analytik Jena, Germany) taking N-acetylglucosamine (GlcNac) as a standard. The enzyme activity was expressed as nmolGlcNac produced min^−1 ^ml^−1^ considering uninoculated medium as blank with three replications. A previously reported pronounced chitinase producer *Streptomyces roseochromogenus* TSR12 [30] was used as positive control (Table 1).

**Table 1:** Quantitative estimation of Antimicrobial and plant growth promoting traits of fluorescent pseudomonads.

Fluorescentpseudomonadsisolates	salicylic acid(µg ml^−1^)	HCN (nmolesmg cellularprotein^−1^)	Chitinase(nmolGlcNacmin^−1 ^ml^−1^)	2,4 DAPG(ng ml^−1^)	PCA(absorbanceat 367 nm)	siderophore(µmol benzoicacid ml^−1^)	Indole(µg ml^−1^) At100 µg ml^−1^tryptophan	ACC deaminase(µmol αketobutyrate mgprotein^−1 ^h^−1^)	Phosphatesolubilization(µg ml^−1^)
GGRJ1	4.093±0.21^f^	22.9±0.81^b^	46.09±1.23^b^	-	-	4.51±0.55^e^	263.33±1.52^e^	-	-
GGRJ2	-	-	-	-	-	-	-	-	4.20±0.11^d^
GGRJ5	-	-	-	-	-	-	328.63±1.49^d^	8.32±1.09^b^	58.84±0.74^b^
GGRJ7	-	-	-	-	-	-	192±2.51^f^	-	-
GGRJ9	-	-	-	-	-	-	158.43±2.37^ab^	-	-
GGRJ12	-	-	-	-	-	-	282.46±2.37^e^	9.51±0.88^b^	-
GGRJ14	11.21±1.07^d^	19.21±0.21^b^	22.77±2.20^c^	0.64±0.09^b^	6.03±0.35^b^	2.62±0.57^e^	466.9±1.41^b^	-	-
GGRJ15	-	-	-	-	-	-	86.8±1.36b^c^	12.28±1.22^a^	-
GGRJ18	-	-	-	-	-	-	97.13±1.01b^c^	5.51±0.95^c^	-
GGRJ19	-	-	-	-	-	-	574.54±1.67^a^	-	-
GGRJ20	9.19±0.67^d^	18.37±0.95^b^	42.70±1.54^b^	-	-	9.48±0.57^c^	-	-	-
GGRJ21	20.50±0.62^a^	30.76±1.07^a^	69.33±1.52^a^	0.863±0.06^a^	6.8±0.49^a^	17.22±0.33^a^	591.14±1.06^a^	14.21±0.41^a^	88.4±1.50^a^
GGRJ22	10.43±1.56^d^	7.27±0.13^c^	-	0.256±0.05^d^	1.43±0.4^d^	5.35±0.25^d^	-	-	-
GGRJ23	4.16±0.80^f^	3.80±0.96^d^	6.56±1.07^e^	0.616±0.05^b^	5.63±0.4^b^	3.23±0.75^e^	75.34±1.02b^c^	-	-
GGRJ24	-	-	-	-	-	-	336.5±1.34^d^	-	-
GGRJ25	7.09±0.58^e^	15.58±0.73^b^	50.44±1.99^b^	0.816±0.06^a^	-	6.53±0.21^d^	437.2±2.15^c^	-	-
GGRJ27	12.14±1.06^d^	17.43±0.51^b^	15.94±1.20^d^	0.5±0.01^c^	-	3.25±0.25^e^	483.9±1.13^b^	-	-
GGRJ29	-	-	-	-	-	-	585.13±1.8^a^	-	-
GGRJ30	14.48±1.63^c^	7.98±1.12^c^	-	0.34±0.04^d^	2.56±0.55^c^	13.81±0.70^b^	-	-	66.66±1.52^b^
GGRJ31	-	-	-	-	-	-	262.3±1.67^e^	-	-
GGRJ33	16.94±0.65^b^	14.61±0.85^b^	-	0.27±0.07^d^	-	14.77±1.15^b^	213.9±2.56^f^	-	-
GGRJ34	3.18±0.23^f^	16.35±1.34^b^	3.40±0.61^e^	-	-	7.10±0.18^d^	-	-	-
GGRJ35	5.27±1.62^e^	9.52±0.56^c^	-	0.16±0.05^e^	1.56±0.4^d^	5.54±0.89^d^	145.6±2.25^ab^	-	46.13±0.96^c^
GGRJ36	15.97±0.36^b^	26.39±0.82^a^	41.27±1.12^b^	0.47±0.06^c^	3.37±0.41^c^	2.39±0.16^e^	183.77±1.63^f^	-	-
GGRJ39	-	-	-	-	-	-	312.94±2.10^d^	-	-
GGRJ42	-	-	-	-	-	-	436.9±1.65^c^	-	-
GGRJ43	-	-	-	-	-	-	326.83±1.68^d^	-	-
GGRJ46	2.58±0.73^f^	5.66±1.07^d^	-	0.21±0.06^e^	-	3.86±0.22^e^		-	-
GGRJ51	-	-	-	-	-	-	455.56±2.65^b^	-	-
GGRJ52	-	-	-	-	-	-	-	-	34±2^c^
GGRJ62	13.4±0.69^c^	3.58±0.52^d^	9.43±0.94^e^	0.2±0.04^e^	-	4.43±0.28^e^	-	-	-
GGRJ63	16.94±1.42^b^	6.47±0.76^d^	7.78±0.61^e^	-	-	11.61±0.81^b^	-	-	43.76±1.56^c^
GGRJ66	3.88±0.39^f^	12.9±0.64^c^	35.48±1.07^c^	0.35±0.08^d^	-	8.89±0.11^c^	-	-	-
GGRJ67	16.87±0.50^b^	10.48±1.15^c^	-	-	-	13.29±0.51^b^	-	-	-
GGRJ68	3.89±0.40^f^	2.16±0.35^e^	-	-	-	6.05±0.75^d^	-	-	14±1.73^d^
GGRJ70	17.7±1.05^b^	26.28±1.14^a^	30.80±1.40c	-	-	4.21±0.94^e^	-	-	-
GGRJ71	-	-	-	-	-	-	-	-	12.11±0.40^d^
GGRJ76	-	-	-	-	-	-	-	-	35.68±0.35^c^
KFP1	6.25±0.96^e^	0.63±0.09^e^	-	0.56±0.12^b^	-	5.73±0.24^d^	429.5±3.1^c^	-	-
KFP2	4.88±0.79^e^	4.69±0.56^d^	-	-	-	11.31±0.85^b^	423.86±2.4^c^	-	-
KFP3	13.13±1.10^c^	1.91±0.23^e^	-	-	-	12.88±1.24^b^	-	-	-
KFP7	16.24±0.85^b^	5.90±1.1^d^	-	-	-	14.35±0.78^b^	-	-	-
*Streptomyces roseochromogenus* TSR12			72.13±1.28^a^						

Means within a column sharing same superscript are not significantly different according to Turkey’s test at p = 0.05; ± means standard deviation (SD); - means no activity.

#### Siderophore Production

Production of siderophore was screened through the chrome azurol S agar (CAS) assay [31]. The hydroxamate nature of siderophore was further detected by Neilands spectrophotometric assay [32]. Quantification was done as described earlier by Reeves et al. 1983 [33]. The absorbance for dihydroxyl phenols was read in a spectrophotometer (Specord 200, Analytik Jena, Germany) at 700 nm. A standard curve was drawn with dihydroxy benzoic acid, and the quantity of siderophore synthesized was expressed as µmol of benzoic acid per ml of culture filtrate. The whole experiments, i.e. CAS assay as well as quantitative estimation was repeated individually for three times to avoid experimental errors.

#### Assay for in vitro salicylic acid (SA) production

Apart from the above antimicrobial traits, SA was estimated to study the indirect mechanism for disease suppression by the *Pseudomonas* isolates. SA triggers plant defense responses by stimulating induced systemic resistance (ISR) in the host plant. Forty eight hours old bacterial culture grown in King’s B broth (KB) was centrifuged at 2800×*g* for 20 minutes at 4°C. Supernatants were acidified to pH 2.0 using 1 N HCl and filtered through nylon membrane under vacuum. Filtrates were partitioned twice with chloroform and ultimately dried under nitrogen steam at 40°C. Samples were analyzed (µg SA ml^−1^ bacterial culture) using HPLC (Waters, Milford, USA) after dissolving them in 23% methanol in 20 mM sodium acetate buffer of pH 5.0 [34]. The whole experiment was carried out with three replications.

#### Detection of Pseudomonas Antibiotic genes

Since, the antibiotics 2, 4-diacetylphloroglucinol (DAPG), pyoluteorin (PLT) and different phenazine (Phz) derivatives have been described in biocontrol *Pseudomonas* spp. as the main cause of their antagonistic activity [35], [36], we further screened for their responsible genes within my antagonistically potential isolates. PCR detection for *DAPG*, phenazine-1-carboxylic acid (*PCA*) and *PLT* was performed as mentioned by Mavrodi et al. 2001 and Raaijmakers et al. 1997 [37], [38]. Further, extraction and phenotypic assays of 2, 4-DAPG and phenazine were carried out as mentioned by earlier workers [10], [39], [40]. PLT was extracted as described by Sarniguet et al. 1995 [41]. TLC was carried out on silica gel G60 to purify the extracted antibiotics. Activated plates were developed at 110°C for 20 minutes were spotted with an ethanolic solution of standard antibiotic (0.5 µg) and 20 µl of the extract running the samples using different mobile phases. Chloroform-methanol 9∶1 (v/v) solvent system was used for DAPG and PCA; however for PLT, chloroform-acetone 9∶1 (v/v) solvent system was used. The corresponding spots by PCA, and DAPG were detected by UV irradiation at 254 nm [21]. PLT spots were detected by spraying with an aqueous 0.5% (w/v) Fast Blue RR salt solution (Sigma Aldrich, USA). Further, antifungal activity of the purified compounds was performed by agar well diffusion assay [28].

### Quantitative Assay of Plant Growth Promoting Traits

#### Estimation of indoles

Pure bacterial cultures were inoculated in DF salts minimal medium [42] with L-tryptophan of different concentration (0, 50, 100, 200 and 500 µg ml^−1^). Bacterial cultures were grown for 48 hours and harvested by centrifugation (4,000×*g* for 20 min at 4°C). Screening of indole production was carried out by mixing the supernatant with Salkowski’s reagent (50 ml, 35% perchloric acid and 1 ml 0.5 M FeCl_3_) in the ratio of 1∶4 (supernatant: reagent) at room temperature (28°C) for 20 minutes. Development of pink color indicated production of indoles. Indole production was quantified by spectrophotometric absorption (Specord 200, Analytik Jena, Germany) at 535 nm with three replications [43]. Standard curve was prepared by using pure IAA (Sigma Aldrich, USA).

#### ACC deaminase Production

Fluorescent pseudomonads were grown in DF salts minimal medium [42] supplemented with 10 µg of ACC (Sigma-Aldrich, USA). ACC deaminase activity was quantified colorimetrically (Specord 200, Analytik Jena, Germany) as discussed earlier and expressed as α-ketobutyrate produced mg of protein^−1 ^h^−1^ [44]. The whole experiment was carried out in three replicates.

#### Quantitative Estimation of Soluble Phosphate

Efficiency of the fluorescent pseudomonads as phosphate solubilizer (PSB) was screened by standard protocol using modified Pikovskaya agar (PKA) medium (gl^−1^ 0.5, yeast extract; 10, dextrose; 5, aluminium phosphate; 0.5, ammonium sulphate; 0.2, potassium chloride; 0.1, magnesium sulphate; 0.0001, manganese sulphate; 0.0001, ferrous sulphate; final pH 3±2) as growth medium. Since use of tricalcium phosphate (TCP) was reported as relatively weak and unreliable factor for isolation of phosphate solubilizing bacteria; for isolation of PSBs from acidic soil of Assam, aluminium phosphate (AlPO_4_) was used replacing TCP in PKA [45]. Clear zone was observed around the bacterial colony after 5 days of incubation at 30°C and portrayed phosphate solubilization by the organism. Quantification of phosphatase activity was carried out as mentioned by Fiske and Subbarow, 1925 with three replications [46].

#### Green house experiment for biocontrol activity

In vivo biocontrol activity of *P. aeruginosa* GGRJ21 againt *Rhizoctonia solani* was performed as described earlier by Saikia et al. 2011 [47]. *Rhizoctonia solani* is the causal organisms of root rot disease of green gram. Soil from green gram cultivated area was collected and autoclaved. Earthen pots were filled up with 5 kg of sterilized soil. Green gram (*var K851*) seeds were surface sterilized with 2% sodium hypochlorite for 30 s, subsequently rinsed with sterile distilled water (SDW) and dried with sterilized air stream. Ten seeds per pot were sown in the previously prepared pots. Twenty days old plants were considered for in vivo experiments. The whole experiment was carried out with six different experimental conditions i.e. (i) pathogen alone (2×10^2^ spore ml^−1^, 5 ml pot^−1^), (ii) GGRJ21 alone (1×10^8 ^CFU ml^−1^, 5 ml pot^−1^), (iii) simultaneous inoculation of pathogen plus GGRJ21, (iv) pre-inoculation of GGRJ21 and then pathogen inoculatation after 2 days (v) post inoculation of GGRJ21 after 2 days of pathogen inoculation and (vi) control (sterile distilled water treatment). Bacterial as well as the pathogen inoculums were prepared as mentioned by Saikia et al. 2011 [47]. Experiment was carried out as complete randomized block design (CRD) with 10 replications. Five microlitres of fungal and bacterial inoculums were applied to each pot. Disease severity was assessed 21 days post inoculation (either pathogen or GGRJ21). The severity of root rot was visually scored by assessing necrotic lesions on the roots and hypocotyls using a rating scale of 0–5 described earlier by Filion et al. 2003 [48].

#### Bacterial growth under water stress condition

Bacterial growth was monitored in 250 ml of nutrient broth (NB) (gl^−1^ 5, peptic digest of animal tissue; 5, sodium chloride; 1.5, beef extract; 1.5, yeast extract; final pH 7.4±2) medium with different water potentials (−0.05, −0.15, −0.30, −0.49, and −0.73 M Pa). The desired osmotic stress condition was developed in the growth medium by adding appropriate amounts of polyethylene glycol (PEG 6000) [49]. One millilitre of overnight grown culture (1×10^9 ^CFU ml^−1^) was inoculated to the prepared NB and incubated at 30°C for 24 hours with continuous agitation of 120 rpm. Bacterial growth kinetics was further recorded colorimetrically (Specord 200, Analytik Jena, Germany) by measuring absorption (600 nm) as a function of time with three replications for each bacterium.

### RNA Isolation and Two Steps Real Time PCR

Fluorescent *Pseudomonas* isolate GGRJ21 was grown under different osmotic conditions (−0.30, −0.49, and −0.73 MPa) [49]. Bacterial cells were harvested in log phase (OD approx. 2.10 at 600 nm). Bacteria grown in normal condition in NB medium were taken as control. Total RNA was isolated from ∼1×10^9^ cells using Geneipure™ Bacterial total RNA isolation kit (Genei, Bangalore, India) as per the manufacturer instruction. One microgram of each RNA sample was reverse transcribed to cDNA with 2X Verso cDNA synthesis kit (Thermo Scientific, USA) using random hexamers. Quantitative amplification reactions of cDNAs from reference genes and target genes were carried out on StepOnePlus™ Real Time PCR System (Applied Biosystems, USA) using Thermo DYNAMO™ 4C SYBR Green qPCR Kit (Thermo scientific, USA). The reaction conditions were set as follows: 10 min at 42°C; 10 min at 95°C; 40 cycles of cDNA amplification for 15 s at 95°C, 30 s at 60°C, 30 s at 72°C with fluorescent signal recording. At the end, a final step of 15 s at 95°C, 1 min at 60°C and fluorescence measured at each 0.7°C variation (from 60°C to 95°C) was included to obtain the melting curve. Four reference genes; *gyrA* (DNA gyrase subunit A), *gmk* (guanylate kinase), sigma factor RpoD (*rpoD*) and *16S rRNA* was selected for normalization of real time PCR reaction. Bacterial osmotic stress responsive genes, *i.e., acdS* (encoding ACC deaminase), *katA* (encoding for catalase) and *gbsA* (encoding for glycine betain) were selected as target genes. The sequences of the genes studied were obtained from NCBI GenBank and the primers were designed with the aid of the OLIGO software (version 5.0; Molecular Biology Insights). The sequences and other properties of the primers are shown in Table 2. Triplicate reaction was maintained for each gene.

**Table 2:** Primers for real time PCR: sequences, final concentration, product size, T_m_ and Efficiency of PCR amplification.

Target	Forward primer(5′-3′)	Reverse primer(5′-3′)	Finalconcentration(pmol µl^−1^)	Productsize (bp)	T_m_	PCR efficiencyvalue(E±SD)
*gyrA*	GACATGATCCCGGAAGAAGA	CAGGTGGGCAATGTAGTCCT	10	150	59	1.963±0.042
*gmk*	CACGACCTGATCCTGGAAAT	TCAATGATCTCGTCGCTGTC	10	152	59	1.857±0.052
*rpoD*	CGAAACGATCAACAAGCTCA	TCACCCAGATGGGAGTCTTC	10	192	60	1.923±0.038
*16S rRNA*	CTCTAAGGAGACTGCCGGTG	CGGACTACGATCGGTTTTGT	10	158	60	2.211±0.056
*acdS*	GCCTCTTCTTCGATACCGTG	AATCATCTCGGCGGTGTTAC	10	179	59	2.012±0.049
*katA*	AAGACCGATATGTTCCTGCG	GTTGAGATCGGGGAACTTGA	10	180	60	1.977±0.044
*gbsA*	GTGCTGACCCTGCAGGTATT	CCTCGAATTCGTCGTAGACC	10	184	60	1.983±0.051

T_m_ means melting temperature; ±SD means standard deviation of the mean value.

### Data Analysis

ARDRA as well as RISA results were analyzed by considering, the character state “1” for clearly detected bands in the gel track and assigned “0” if it was absent or impossible to determine. The data matrix was generated by Jaccard’s similarity coefficient algorithm. Each pair-wise comparison was constructed from the similarity matrix by the un-weighted pair group method with arithmetic mean (UPGMA). For rep-PCR (BOX-PCR and ERIC-PCR) fingerprinting analysis, the photographs were imported into the software package BioNumerics version 2.5 (Applied Maths, Belgium). Similarity matrices from densitometric curves of the gel tracks were calculated using the Pearson’s product moment correlation coefficient followed by dendrogram construction using UPGMA algorithm. The *16S rRNA* gene sequences of our isolates along with their closest homolog were aligned in Clustal W, where a total ninety (*16S rRNA* of 85 bacterial isolates from the present study and 5 reference sequences from GenBank) sequences were considered. Both distance-based and character-based method was used for inferring the phylogenetic relationship among various FPs. For distance based method Neighbor-Joining (NJ) algorithm was employed whereas for character-based method Unweighted Pair Group with Arithmetic Mean (UPGMA) algorithm was employed. In both the cases Kimura-2-parameter substitution model [50] was employed. The robustness of the inferred phylogenetic trees was tested by bootstrap analysis [51] with 1000 iterations of the original dataset. The triplicate data generated during quantitative evaluation of antimicrobial, and PGPR traits were analyzed by means of one-way ANOVA and means were compared by the Tukey’s test, using the SPSS software (ver. 10.1, SPSS Inc., www.spss.com) at the significance level *p* = 0.05. The sequences of isolates in the present study were submitted to GenBank of NCBI and obtained accession numbers. During quantitative reverse transcription PCR, tenfold serial dilution of cDNA curves were produced to calculate the amplification efficiency for all genes through the equation E = 10^(−1/slope)^ [52]. Threshold cycle (C_T_) was compared with log_10_ relative copy number of the sample from a dilution series. Normalization of PCR reaction with four different reference genes was performed. Relative expression level obtained for target genes were compared when three candidate normalizer genes were used individually. Then, the best combination was obtained by geNorm software [53]. Expression levels were determined as the number of cycles needed for the amplification, to reach a threshold fixed in the exponential phase of PCR reaction (C_T_) [54]. C_T_ values from the ABI Step one plus System (Applied Biosystems, Foster City, California, USA) were analyzed through 2^–ΔΔC^_T_ method, where ΔΔC_T_ = ΔC_T_sample–ΔC_T_control [55]. The green house experimental data were analyzed by LSD test at P<0.05 using Duncan’s multiple range tests.

## Results

### Isolation and Phenotypic Characterization of the pseudomonad isolates

Aerobic incubation of soil suspension from lower dilution series in different *Pseudomonas* specific media for 24 hrs at 30±2°C resulted in 437 colonies of distinct morphotypes. The initial screening through UV light (λ = 356 nm) revealed that only 120 were fluorescent *Pseudomonas* out of total 437 bacterial isolates. Microscopic observation showed that all the isolates were rod shaped, motile and gram negative. All the isolates showed positive reaction for catalase, oxidase, and gelatine liquefaction. The biochemical characteristics of the isolates are summarized in Table S1.

### Rep PCR Fingerprinting

The BOX PCR fingerprinting revealed a banding pattern from 200 to 5000 bp, similarly for ERIC-PCR, the molecular weights of the amplified products were estimated at approximately 100–5500 bp among the 120 pseudomonad isolates. A total of 25 unique rep-PCR fingerprints were detected among the strains isolated during the vegetative-growth as well as reproductive phase of the crop (Fig. S1). The dendrogram showed two major clusters; one large (Cluster-I) and one small (Cluster-II) (Fig. S2). Both the clusters attained a similarity coefficient value of 75%. Further, cluster I is sub-divided into nine distinct sub-clusters. The sub-cluster-Ia is formed of 3 isolates with a coefficient value of 88%, whereas its closest sub-cluster-Ib is comprised of 54 isolates shared approximately 95% similarity coefficient. Sub-cluster-If with 48 members constituted the next large group after Ib. Most of the members of sub cluster Ib were isolated during the reproductive phase of the pulse crop. All the members of sub-cluster-Id and If were obtained while the crop was in vegetative stage. The isolates from GGRJ86-GGRJ120 (subcluster If) were 100% identical to each other.

### Amplified Ribosomal DNA Restriction Analysis (ARDRA)

Restriction digestion profiles of *16S rRNA* amplicons with *Hae*III, *Alu*I and *Msp*I on 2% agarose gels were compared to avoid redundancy among the strains. According to the type of restriction enzyme, the *16S rRNA* fragment patterns were different, and showed notable genetic differences within the microbial communities. *Hae*III showed 17 restriction patterns; whereas *Alu*I and *Msp*I revealed 9 and 20 restriction patterns, respectively (Fig. S3a). Based on ARDRA fingerprinting with three endonucleases, dendrogram was constructed. The resulted dendrogram can be divided into two major clusters (Fig. S4). The large cluster-I formed three distinct sub-clusters (Ia, Ib and Ic). The sub-cluster Ic comprises of maximum number of isolates sharing the similarity coefficient of 76%. All the members from Ic were isolated from reproductive growth phase of green gram, representing high bacterial diversity. Interestingly, two pseudomonad strains GGRJ1 and GGRJ2 isolated during the reproductive phase of the pulse were out-clustered from the other and grouped in a separate sub-cluster Ia. The sub-cluster Ib with 24 numbers of FP shared 78% similarity with rest of the bacterial strains. Most of the isolates of sub-cluster Ib with were associated with reproductive phase of the pulse crop. Most of the members from sub-cluster Ic representing poor genetic diversity were related to vegetative phase of the crop. Pseudomonad isolates from GGRJ86 to GGRJ120 were under Cluster II and shared closest genetic similarity that correlates with the findings from rep PCR genotyping. Majority of isolates from II were associated with vegetative phase of the pulse crop.

### Ribosomal Intergenic Space Analysis (RISA)

Comparative analysis of the 16S–23S rRNA intergenic space indicated a higher degree of genetic diversity among the isolates (Fig. S3b and S5). The dendrogram showed the formation of two large clusters at 70% similarity coefficient. Cluster I can be divided to two sub-clusters (Ia and Ib). Both the sub-clusters Ia and Ib included almost equal number of isolates. All the members of sub-cluster Ib were associated with vegetative growth phase of green gram showed close affinity among the isolates from GGRJ86 to GGRJ120. Cluster II seemed to be somehow different from cluster I comprising 17 isolates with three distinct sub-clusters depicted a significant genetic diversity among them.

### Molecular Phylogenetic Analysis

**Figure 1:**
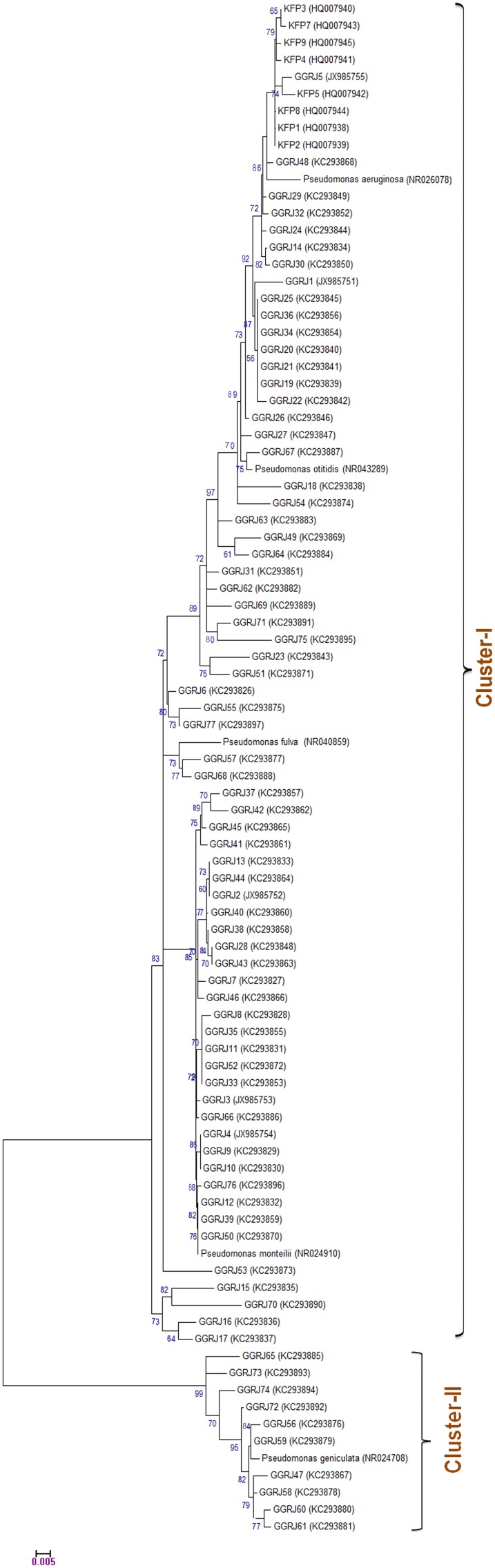
Phylogenetic analyses of fluorescent pseudomonads based on the nucleotide sequence of *16S rRNA*. The multiple sequence alignment was done in CLUSTALW program embedded in MEGA version 5.10. The pair-wise evolutionary distances were calculated using Kimura-2 parameter model. The phylogenetic tree was constructed by Neighbor-Joining (NJ) method with 1000 replicates using bootstrap. A total of 5 reference fluorescent pseudomonad strains were used for the tree construction. Bar, .0.005 shows the substitutions per nucleotide position.

Based on the genotypic analysis, 35 closest fluorescent pseudomonads (GGRJ86 to GGRJ120) were excluded from further molecular phylogenetic study. All of them showed close affinity to *P. aeruginosa*. *16S rRNA* sequence of rest 85 isolates was compared against NCBI nucleotide database and confirmed the presence of 23 different species of pseudomonads (Table S1). Moreover, it was found that most of the isolates shared highest percentage of sequence identity with that of *P*. *aeruginosa*. Phylogenetic relatedness among the 85 FP isolates was established through NJ and UPGMA method. For proper comparison, five reference sequences from NCBI genebank database were used during the study. Both NJ and UPGMA methods yielded same type of tree topology with two major clusters (Fig. 1 and Fig. S6). As evidenced from the Fig. 1 that the larger cluster *i.e.*, cluster-I consist of 75 numbers of isolates with three reference bacteria (*i.e.*, *P. aeruginosa* strain DSM 50071 (NR026078); *P. monteilii* strain CIP 104883 (NR024910) and *P. fulva* strain AJ 2129 (NR040859)) at 70% cut off value. The reference strain, *P. aeruginosa* formed largest clusters with its closest relatives representing the major group,. All the *Pseudomonas* isolates of cluster-II conferred their placement in Gamma-β proteobacteria group along with the reference strain (*P*. *geniculata* strain ATCC 19374 (NR024708). Further, the placement of various clades in distinc phylogenetic position from NJ clustering was compared with UPGMA clustering (Fig. S6) which conferred correct phylogenetic position of each isolate.

### Antimicrobial Activity

Bacterial secondary metabolites were tested against the plant pathogenic fungi *Rhizoctonia solani*, *Fusarium oxysporum* f. sp. *raphani*, *F. oxysporum* f. sp. c*iceri* and *F. semitectum*. Out of 120 bacterial isolates, 23 isolates exhibited mixed antagonistic activity against the pathogens. The strain *P. aeruginosa* GGRJ 21 was found to be the most prominent, showing activity against all the fungal pathogens (Table 3). After GGRJ21, antifungal activity of GGRJ1, GGRJ14, GGRJ20, GGRJ25, GGRJ27, GGRJ36, GGRJ70 and KFP2 were found to be promising. GGRJ21 exhibited pronounced antagonistic activity against *R*. *solani*, one of the important fungal pathogen of green gram with 23 mm of inhibition zone.

**Table 3:** Antagonostic activity of fluorescent pseudomonads against phytopathogenic fungi. Activity was monitored on the basis of inhibition zone.

Sl. No.	*Pseudomonas* isolates	Zone of inhibition (ZOI) (mm)			
		*R. solani*	*F. oxysporum f. sp. raphani*	*F. oxysporum f. sp. ciceri*	*F. semitectum*
1	GGRJ1	17±1^a^	12±1^a^	10±0.76^a^	-
2	GGRJ14	12±1.21^b^	13±1.56^a^	-	10±0.77^a^
3	GGRJ20	-	17±0.65^b^	14±1.2^b^	12±1^a^
4	GGRJ21	23±0.83^c^	19±1^b^	21±1.11^c^	16±1.42^b^
5	GGRJ22	-	-	-	12±0.43^a^
6	GGRJ23	17±1^a^	-	14±0.95^b^	-
7	GGRJ25	14±1.54^b^	9±1.41^c^	-	15±1.3^b^
8	GGRJ27	16±1^a^	-	14±1^b^	8±0.2^c^
9	GGRJ30	10±1.1^d^	-	-	7±0.76^c^
10	GGRJ33	-	14±1^a^	-	7±0.28^c^
11	GGRJ 34	-	7±0.86^c^	13±1.13^b^	-
12	GGRJ35	-	-	-	7±0.65^c^
13	GGRJ36	-	8±0.78^c^	20±1.24^c^	6±0.73^c^
14	GGRJ46	-	8±1^c^	-	-
15	GGRJ62	-	-	9±0.76^a^	-
16	GGRJ63	-	12±0.64^a^	8±1.21^d^	-
17	GGRJ66	-	-	18±1.54^c^	12±1.42^a^
18	GGRJ67	-	8±1.12^c^	-	-
19	GGRJ68	-	-	9±0.54^a^	-
20	GGRJ70	-	7±0.76^c^	20±1.32^c^	6±0.76^c^
21	KFP1	8±1^d^	14±1^a^	-	13±1^a^
22	KFP2	-	8±0.65^c^	7±1^d^	15±1.3^b^
23	KFP3	-	-	6±0.67^d^	7±0.43^c^

Means within a column sharing same superscript are not significantly different according to Turkey’s test at p = 0.05; ± means standard deviation (SD) and - means no activity.

### Antimicrobial Traits

Variable production of HCN, chitinase, siderophore and salicylic acid by the 23 isolates were determined by standard protocols. GGRJ21 was found to be excellent in production of antimicrobial traits amongst the 23 isolates (Table 1). The intrinsic ability to produce HCN was varied greatly among the antagonistically potential isolates. Values were expressed as nmoles mg^−1^ cellular protein, varied greatly from 0.72 to 30. Extracellular chitinase production was recorded in 13 isolates. Highest chitinase production was monitored in strains GGRJ21, followed by GGRJ25, GGRJ1, GGRJ20 and GGRJ36. Similarly, a distinct variation in siderophore production was monitored among the isolates; where GGRJ21 again proved its superior nature with a production efficacy of 17.2 µmol benzoic acid ml^−1^. Highest production of salicylic acid by GGRJ21 was calculated as 20.5 µg ml^−1^, followed by GGRJ70, GGRJ33, GGRJ67 and KFP7 having the production efficiency of 17.7, 16.9, 16.8 and 16.2 µg ml^−1^ respectively.

Detection of the antibiotic coding genes for 2, 4-diacetylphloroglucinol (*DAPG*), phenazine-1-carboxylic acid (*PCA*) and pyoluteorin (*PLT*) from bacterial genomic DNA were conducted by using gene-specific primers within the 23 antagonistically potential *Pseudomonas* isolates. PCR amplification reaction with the nucleotide primers Phl2a and Phl2b revealed amplification of 745-bp fragment of the 1,001-bp *phlD (DAPG)* gene in the 14 isolates (GGRJ14, GGRJ21, GGRJ22, GGRJ23, GGRJ25, GGRJ27, GGRJ30, GGRJ33, GGRJ35, GGRJ36, GGRJ46, GGRJ62, GGRJ66 and KFP1). The phenazine biosynthetic gene cluster contains 7 genes, of which five (*phzC-G*) are essential and two others (*phzA* and *B*) substantially enhancing the level of synthesis of phenazine-1-carboxylic acid (PCA). The Primers PCA2a and PCA3b amplified a region of 1150 bp (within *phzC* and *phzD*) in 7 bacterial isolates (GGRJ14, GGRJ21, GGRJ22, GGRJ23, GGRJ30, GGRJ35, and GGRJ36). Pyoluteorin (PLT) biosynthesis linked with the gene cluster of 10 genes (*pltL, pltB, pltC, pltE, pltF, pltG, pltA, pltD, pltM* and *pltR*). PCR screening revealed the presence of *pltB* gene (type I polyketide synthases) in the single pseudomonad isolate, GGRJ21 with a product size of 779 bp. The phenotypical observation showed varied efficiency of 2, 4 - DAPG production among the 14 *phlD* positive isolates. *Pseudomonas* isolate GGRJ21 showed maximum production efficiency (0.863****ngml^−1^) of 2, 4 - DAPG (Table 1). Similarly, varied degree of phenazine production was observed. *Pseudomonas* isolate GGRJ21 was again marked as dominant antibiotic producer than rest of the isolates (Table 1). The purified antibiotic extracts, *i.e*. *PLT* and *PCA* revealed a significant antibiosis against the phytopathogens except DAPG (Figure S7).

### Plant Growth Promoting Traits

Out of 120 numbers of isolates, production of indoles, ACC deaminase and phosphatase activity were found to occur in 25 (20.8%), 5 (4.1%), and 10 (8.3%) numbers of fluorescent isolates respectively (Table 1). GGRJ21 showed higher level of indole production (591.14 µg ml^−1^ at 100****µg ml^−1^ tryptophan concentration) among the isolates. The indole production was recorded maximum at 100 µg/ml tryptophan concentration; while a gradual decrease was recorded at higher tryptophan concentration (Table S2). GGRJ21 was again dominant producer of ACC deaminase and phosphatase with quantitative estimation values of 14.2 µmole α-KBh^−1 ^mg protein^−1 ^h^−1^ and 88.4****µg ml^−1^ phosphate solubilized.

### Green House Experiment

Red lesions on hypocotyls and tap root system of the green gram seedlings clearly indicated the deleterious attack of *R. solani* on the host plant. However, the rate of disease severity was less in the GGRJ21 treated plants as compared to the plant samples with the pathogen alone with significantly (p = 0.05) less number of lesions (Table 4). GGRJ21 suppressed root rot disease of green gram by 28–93%. Control healthy plants without any inoculation and seedlings with GGRJ21 alone did not show any disease symptoms. Disease severity in GGRJ21 pre inoculated seedlings was significantly different (p = 0.05) with low disease rate as compared to other treatment condition.

**Table 4:** Effect of *Pseudomonas aeruginosa* GGRJ21 on root rot disease suppression of green gram during infection with *Rhizoctonia solani*.

Treatments	Disease severity		
	Trial 1	Trial 2	Trial 3
Control (sterile distilled water treatment)	0	0	0
Pathogen alone	5.17±0.351^a^	4.93±0.4^a^	5.13±0.55^a^
GGRJ21 alone	0	0	0
Simultaneous inoculation of pathogen plus GGRJ21	2.99±0.11^b^	2.87±0.28^b^	2.81±0.17^b^
Pre-inoculation of GGRJ21 and then pathogen was inoculated after 2 days	1.14±0.15^c^	1±0.2	1.11±0.22^c^
Post inoculation of GGRJ21 after 2 days of pathogen inoculation	3.69±0.16^b^	3.67±0.67^b^	3.51±0.09^b^

Means within a column sharing same superscript are not significantly different according to Turkey’s test at p = 0.05; ± means standard deviation (SD).

### Bacterial Growth Kinetics under Osmotic Stress

Though ten numbers of isolates from drought prone rhizosphere microhabitat showed growth under osmotic stressed condition; however at high stressed condition (−0.30, −0.49, and −0.73 M Pa) only *Pseudomonas* isolate GGRJ21 showed vigorous growth among them (Table S3). Twenty hours observation under osmotic stressed condition clearly emphasized the capability of GGRJ21 to survive under extreme stressed condition.

### Relative Quantification of Stress Responsive Genes

Among the four housekeeping genes; *gyrA*, *gmk*, *rpoD* and *16S rRNA; 16S rRNA* was verified to have the lowest average expression stability (M) when samples experiencing osmotic stress were analyzed (data not shown). The relative expression level of *acdS*, *katA* and *gbsA* gene transcripts in GGRJ21 were analyzed over four treatment condition; A, B, C, D (where, A-GGRJ 21 grown in normal NB medium, B-GGRJ21 grown in –0.3****mPA, C-GGRJ21 grown in 0.49****mPA and D-GGRJ21 grown in –0.73****mPA). Sample A was used as calibrator for all the three experiments. Thus the C_T_ value obtained in sample A was taken as the control value in order to calculate the fold change in gene expression over each of the three samples.

**Figure 2:**
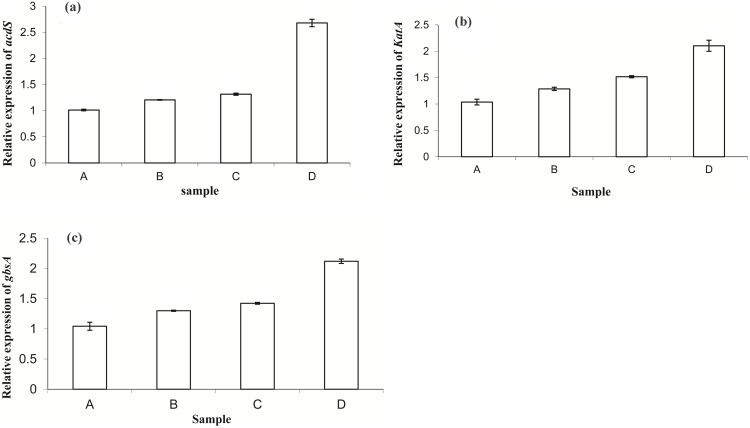
Relative gene expression level of, a) *acdS*, b) *katA* and c) *gbsA* in *P*. *aeruginosa* (GGRJ21) growing in different osmotic stress condition. A-GGRJ 21 grown in normal NB medium, B-GGRJ21 grown in –0.3****mPA, C-GGRJ21 grown in 0.49****mPA and D-GGRJ21 grown in –0.73****mPA.

The transcripts of the *acdS*, *katA* and *gbsA* gene from bacterial mass growing under different conditions were detected. Transcript copy numbers of each gene were found to be in increasing order for bacterial samples growing in lower to higher osmotic stressed condition (Fig. 2a, b, c). Thus *acdS*, *katA* and *gbsA* showed significant up-regulation in the bacterial cells after a gradual increase of osmotic stress from −0.3****mPA to −0.73****mPA. Almost 3 fold increase in expression level of *acdS* was noticed, which was the best in comparison to the other two target genes, *i.e.*, *katA* and *gbsA*. Moreover, highest transcript copy numbers for each case was recorded for cell cultures growing under −0.73****mPA. Overall, the transcriptional activity of the three target genes up regulated over a function of increasing osmotic stress condition. A linear relationship was obtained between threshold cycles (C_T_) and the log copy number of cDNA for all genes with an correlation coefficient (*R^2^*) ranging from 0.96 to 0.99, indicating that C_T_ values changed proportionally to the serial dilution of the samples. The E value, within the range 1.857 to 2.211, indicated the efficient amplification near the theoretical optimum level of 2.

## Discussion

Among the plant growth promoting rhizobacteria (PGPR), fuorescent pseudomonads, account for a significant proportion of the culturable rhizosphere population [56]. Since this ubiquitous group possess PGPR as well as biocontrol activities, their occurrence in the crop rhizosphere might be very essential for substantial crop development.

In this present investigation, we examined the genetic diversity and functional characterization of culturable fluorescent pseudomonads isolated from green gram rhizosphere. A comparative analysis was performed between the pseudomonad strains isolated during vegetative as well as reproductive growth phase of the pulse crop. Different PCR-based techniques *i.e.*, Rep PCR ribotyping, ARDRA and RISA, were employed to investigate the genetic variability of fluorescent pseudomonad isolates. Although the different PCR based genotypic analysis tools have their own level of genetic resolution to differentiate the organisms, they were not sufficient to resolve the fine genetic variation of all the isolates in this study. Genetic distance among the isolates within a same cluster was varied significantly depending upon the molecular markers used. For instances, isolates like GGRJ40, GGRJ41, GGRJ43, GGRJ44, GGRJ45, GGRJ46, GGRJ47, GGRJ55, GGRJ63, GGRJ75 and KFP8 were placed in sub-cluster If, Ic and Ia of rep PCR, ARDRA and RISA respectively, but their distant location in the clusters denoted intrageneric diversity within the isolates. In addition, genotypic fingerprints revealed close evolutionary relationships within the FPs isolated from vegetative growth phase of green gram with monophyletic clustering. However, significant diversity was observed in case of isolates linked with reproductive growth phase of the pulse crop, forming para/poly phyletic group among them. The lack of genotypic correlation within the FPs linked with reproductive growth phase of the pulse crop was observed because of the existence of high degree of genetic diversity within the isolates as reported by earlier workers [57], [58].

*16S rRNA* sequence analysis of isolates delineated 85 isolates representing 23 distinct species of FPs. Reconstruction of molecular phylogeny grouped 85 isolates into two major clusters. All the isolates of cluster II were linked to reproductive growth phase of the host plant and showed closest homology to the Gamma-β proteobacteria group. This exception colonization suggests the variation in root microenvironment pattern during reproductive growth phase of the pulse crop. However, cluster-I with 75 isolates depicted predominant occurrence of *P. aeruginosa*, *P. otitidis* and *P. plecoglossicida.* Among the green gram associated FPs, *P. aeruginosa* clearly signifies the dominant chronicle with19 bacterial isolates followed by *P. otitidis*, *P. plecoglossicida, P. monteilii* and *P. mosselii*. Earlier studies conducted by Weisburg et al. 1991 and Widmer et al. 1998 [59], [60] have shown that *P. fluorescens* and *P. putida* are the presumed to be dominant species in rice and wheat rhizosphere. Similarly, studies conducted in rice rhizosphere revealed dominant population of *P. fluorescens*, *P. aeruginosa*, and *P. putida* [61]. Nayak et al. 2008 reported *P. monteilii* as the dominant species in the banana rhizosphere along with other species of fluorescent pseudomonads [13]. Moreover, as compared to other PCR-based genotyping, *16S rRNA* analysis depicted a clear resolution of genetic distance and diversity among the diverse taxa of FPs; as evidenced from phylogenetic trees inferred through NJ and UPGMA method.

Variation in genetic composition between the vegetative and reproductive phase associated FP communities clearly suggests the role of ecological conditions on microbial species distribution; directly correlated with the previous findings [62]–[64]. Earlier studies by McArthur et al.1988 [65] revealed that habitat variability due to change in soil properties could influence the genetic diversity in natural populations of a soil bacterium. Again structural diversities among the microorganisms in the rhizosphere soil may be also due to differences in root exudation during different growth phase, as well as for soil physicochemical properties and agronomic practices [66], [67].

Based on metabolic characteristics, bacterial functional traits were studied extensively. In natural condition different plant growth promoting (PGP) as well as biocontrol traits of root associated microflora plays a crucial role in better plant growth and development [66]. In recent years, much attention has been given to the antagonistic activities and bio fertilizing abilities of fluorescent pseudomonads associated with agricultural crop plants [7]. Antagonistic activity of HCN against different phytopathogens has been already reported [68]. In the present investigation, all the antagonically active FP showed a varied range of HCN production. However, eight strains (GGRJ21, GGRJ70, GGRJ36, GGRJ1, GGRJ14, GGRJ20, GGRJ27 and GGRJ34) with high HCN production capability showed pronounced antagonistic activity against almost all the tested fungal pathogens. Kloepper et al.1980 [69] demonstrated the role of siderophore production as one of the significant characteristics of biocontrol agents. Our observation shortlisted ninety six percent of the *Pseudomonas* isolates with biocontrol activity (23 out of 24) produced siderophore (hydroxamate type) with varying degrees of production efficiency that correlates with the previous report [70]. Establishment of iron delimiting condition in the rhizosphere is the main mechanism of siderophore based inhibition the root colonizing pathogenic fungi [71]., Chitin amendment significantly increased the chitinase activity of *Pseudomonas* strains. Among the thirteen strains, *P. aeruginisa* GGRJ21 responded well to the addition of chitin and produced 69.33 nmolGlcNac/min/ml chitinase in chitin- amended medium. Moderate response to the addition of chitin was noticed in other strains. However, the hydrolytic nature of chitinase is only potential to inhibit or degrade the chitin containing pathogens. Thus, chitin production may not be equally relevant in protecting all types of phytopathogens. It have been established that, majority of PGPR activate induced systemic resisrance (ISR) via a SA-independent pathway involving jasmonate and ethylene signals [72], [73]. Most of the fluorescent pseudomonads with antagonistic activity revealed significant levels of SA production. SA production triggers ISR development in the host plant by controlling the major pathogen related (PR) gene expression, which might be the one proficient path for disease management.

Moreover, wide range of antibiotic production efficiency i.e. DAPG, PCA and PLT by FP isolates may play a significant role in suppressing various soil borne pathogens around the green gram rhizosphere. DAPG are an important group of polyketide antibiotics that may have the capability to suppress root and seedling diseases on a variety of crops [13]. DAPG retains a wide range of antifungal, antibacterial as well as antinematocidal activity. The antagonistic activity of DAPG was recorded against a wide array of fungal pathogens, i.e, *Thielaviopsis basicola* [12], *Gaeumannomyces graminis* [12], *Fusarium oxysporum* f. sp. l*ycopersici* [74], *Fusarium oxysporum f. sp. radicis-lycopersici* [75] etc. Local antagonism in the plant root system and induction of plant defense mechanism may be the main reason for DAPG antagonism against *Thielaviopsis basicola* and *Gaeumannomyces graminis* [12]. Duffy and Defago 1997 [75] stated the role of zinc amendments on enhncement of DAPG production and supression of *Fusarium oxysporum f. sp. radicis-lycopersici* by reducing the fusaric acid production. Saomeya et al. 2007 [74] observed a synergistic effect of DAPG and chitinolytic enzymes against *Fusarium oxysporum* f. sp. l*ycopersici.* However, during the study purified DAPG was found to be ineffective against the fungal pathogen tested. The observation was found to be corroborated with the previous findings of Reddi and Borovkov 1970 [76], where they had enumerated the poor activity of DAPG against fungi and many gram negative bacteria. Thus the result concluded the narrow spectrum activity of DAPG extracted from the fluorescent pseudomonad isolates. Similarly, a number of broad spectrum antifungal colored phenazines of fluorescent pseudomonads origin have been reported in previous studies [77]–[80]. Our present investigation was also reflected the similar predisposition with broad range of antagonistic activity of purified phenazine-1-carboxylic acid against all phytopathogenic fungi tested. During the study, PLT activity was retained by only one isolate, *i.e.* GGRJ21, which showed broad range of antibiotic activity against all the phytopathogens. Broad range of anti microbial activity of purified PLT from fluorescent pseudomonads has been reflected from the earlier investigations [12], [81], [82].

Screening for the production of different biofertlizing attributes from green gram rhizosphere associated FP isolates, depicted a significant variation in the production of a wide array of metabolites and enzymes. A total of 25 numbers of isolates showed indole production. The spectrophotometric assay clearly distinguished gradual increase in IAA production with increase in L-tryptophan concentration from 0 to 100 µg ml^−1^. The result, *i.e* enhancement of L-tryptophan-derived auxin biosynthesis can be directly correlated with the previous findings [83], [84]; *i.e* further amendment of L-tryptophan (beyond 100****µg ml^−1^) negatively stimulated the in vitro level of auxins production by the FP isolates. Thus suggested a negative feedback loop [85]. Although ACC deaminase activity is very prominent in pseudomonads group; during our study, we screened out only five pseudomonad isolates with this particular enzymatic activity. Moreover, the enzyme production efficiency was found to be better than the previous reports [86], [87]. These specific enzyme positive isolates may play a vital role in root elongation and seed germination as reported earlier [88]. During the study, we had screened out all the *Pseudomonas* isolates for their phosphate solubilising activity. However only ten numbers of isolates showed phosphate solubilization with less phosphatase activity than some of the previous reports [18], [89]. Lower proportion of phosphate solubilization might be due to release of lesser concentration of organic acids from the PSBs required to mobilize major quantities of P into the soil solution [90], [91]. However, organic acid production does not seem to be the only mechanism of inorganic phosphate solubilization; as in some cases pH reduction did not correlate with the mineral phosphate solubilization [92]. Release of protons accompanying respiration or ammonium assimilation was reported to be another mechanism for phosphate solubilization [93].

Among the 120 bacterial isolates, *Pseudomonas aeruginosa* GGRJ21 was selected further for in vivo experiments mainly due its wide range of antagonistic, plant growth promotion as well as effective range of stress tolerant propensity. The *in* *vivo* pot experiment with *P*. *aeruginosa* (GGRJ21) and *R*. *solani*, presented a practical and proficient green approach to control root rot disease of green gram. *In vivo* trial in green house condition strongly supported the *in vitro* antifungal activity of GGRJ21. Prior inoculation of GGRJ21, suppressed the rate of disease severity much more efficiently than the control. This may be due to systemic resistance induced by the biocontrol agent in the host plant [94], [95]. However, other possible mechanisms could be through antibiosis [94], mycoparasitism [96] or for competition for nutrients and/or space [97]. The relative biocontrol activity of GGRJ21 could be corroborated with the earlier reports [2], [98]–[100]. However, greater disease suppressive propensity could be enough to promote *P. aeruginosa* GGRJ21 as a most efficient biocontrol agent than the previously reported microbial strains.

The osmotic strength of the environment is one of the major physical parameters that determines survival rate of the organism in their own habitat. Variation in osmotic stress in the environment is an indisputable reality for microbes colonizing any environment [101]. Being an opportunistic organism, pseudomonads acquire up adaptive cellular machinery, which assist them for survival in diverse and often stressful environmental conditions [101]. Several genes differentially expressed during stressed condition, which may play a key role in the prevalence and persistence of the bacterium in osmotically stressful infection sites [102]. Accumulation of *N*-acetylglutaminylglutamine amide and glycinebetaine as cytoplasmic osmoprotectants in response to osmotic stress is one of the important distinctiveness of *P*. a*eruginosa* [103]. According to the earlier workers, transcriptional product of two genes *gbsA* and *gbsB* was essential for glycine betaine biosynthesis in bacteria. Since *P*. *aeruginosa* (GGRJ21), showed stable growth kinetics under optimum osmotic stress condition, we further analyzed the relative expression study of *gbsA*. As would be expected, *gbsA* showed enhanced levels of expression during the early stages of osmotic up-shock. Interestingly, the expression of gene *katA* associated with the catalase was substantially over-expressed in osmotically shocked cells, thus protect the *Pseudomonas* cell from the toxicity of hydrogen peroxide (H_2_O_2_). Further we have analyzed the relative expression study of *acdS* (gene encoding for ACC deaminase) regulation and found almost three fold up-regulation during osmotic shock at −0.73****mPA condition. Plants under water stress experiences high level of ethylene biosynthesis, which thereby causes fatal effect on plant cell. However, the application of PGPR containing ACC deaminase is very crucial agent to regulate the plant ethylene by converting, ACC into α-ketobutyrate and ammonia, thus helps the plant to live under adverse climatic condition. Since *P*. *aeruginosa* (GGRJ21) showed a remarkable synthesis of ACC deaminase due to up-regulation of *acdS* gene under osmotic stress condition, application of the *Pseudomonas* strain may interact with green gram and confer a resistance support to the pulse crop under water deficit condition. Moreover, we have already examined the positive effect of *P. aeruginosa* (GGRJ21) towards the alleviation of drought stress in green gram plant in normal environmental condition [4].

## Conclusion

Knowledge of the PGPR diversity and their bio-control, biofertilizing activity is not only essential to understand their ecological role in the rhizosphere, but also for utilization in sustainable agriculture. The present study showed a high degree of functional and genotypic diversity among fluorescent pseudomonads in the green gram rhizosphere for the first time. *Pseudomonas aeruginosa* GGRJ21 with innate biocontrol, osmotolerant and biofertilizing potential could provide a vital bio-resource for plant growth and development, promotion, disease control, and subsequent enhancement of crop yield in the host plant.

## Supporting Information

Figure S1 **Rep-PCR genomic fingerprints of 120 dominant strains generated with BOX AIR1 (A) and ERIC1 (B) primer with 500 bp DNA marker.**
(DOCX)

Figure S2 **Dendrogram showing the genetic diversity of fluorescent pseudomonads of green gram rhizosphere.** Clustering analysis based on the combined fingerprints of ERIC and BOX-PCR was performed using the UPGMA method followed by Jaccard’s coefficient.
(DOCX)

Figure S3 **Restriction patterns of PCR amplified fragment of (a) 16S rDNA digested with** ***HaeIII*** **,** ***AluI*** **and** ***MspI*** **and (b) 16S–23S rDNA intergenic spacer region digestion with** ***MspI*** **.**
(DOCX)

Figure S4 **Dendrogram showing the genetic diversity of fluorescent pseudomonads of green gram rhizosphere.** Clustering analysis of ARDRA fingerprints was performed using the UPGMA method followed by Jaccard’s coefficient.
(DOCX)

Figure S5 **Dendrogram based on RISA analysis (restriction digestion of 16S–23S rDNA intergenic spacer region sequences by** ***MspI*** **) showing the intra specific relationships among 120 members of the fluorescent pseudomonads.**
(DOCX)

Figure S6 **Phylogenetic analyses of fluorescent pseudomonads based on the nucleotide sequence of** ***16S rRNA*** **using UPGMA method in MEGA 5.2.** The Bar, 0.005, shows the substitutions per nucleotide position.
(DOCX)

Figure S7 **Antifungal activity of purified phenazine-1-carboxylic acid (PCA) and pyoluteorin (PLT) against phytopathogenic fungi.**
(DOCX)

Table S1 **Eighty-five** ***Pseudomonas*** **isolates with their taxonomic, phenotypic and biochemical characteristics.**
(DOCX)

Table S2 **Quantitative estimation of indoles with tryptophan concentrations of 100, 200 and 500 µg ml^−1^.** Values are mean of three replicates.
(DOCX)

Table S3 **Optical density (OD) of 10** ***Pseudomonas*** **isolates at 600 nm after 24 hours of growth under different osmotic stress condition considering normal growth in nutrient broth (NB) as control.** Values are mean of three replicates.
(DOCX)
