# Supplementary figures and images for: Community Profiling of Culturable Fluorescent Pseudomonads in the Rhizosphere of Green Gram (Vigna radiata L.)
Source: PLoS One. 2014 Oct 3;9(10):e108378. doi: 10.1371/journal.pone.0108378 (PMC4184808; doi:10.1371/journal.pone.0108378)

**Fig. S1**

**
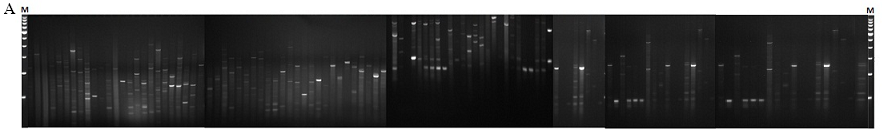
**


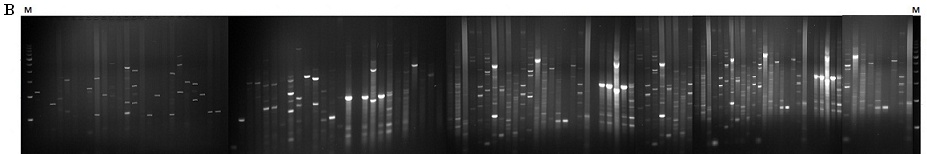

Supplement: Figure S1 — Rep-PCR genomic fingerprints of 120 dominant strains generated with BOX AIR1 (A) and ERIC1 (B) primer with 500 bp DNA marker. (DOCX) [file pone.0108378.s001.docx]

**Fig. S2**

**
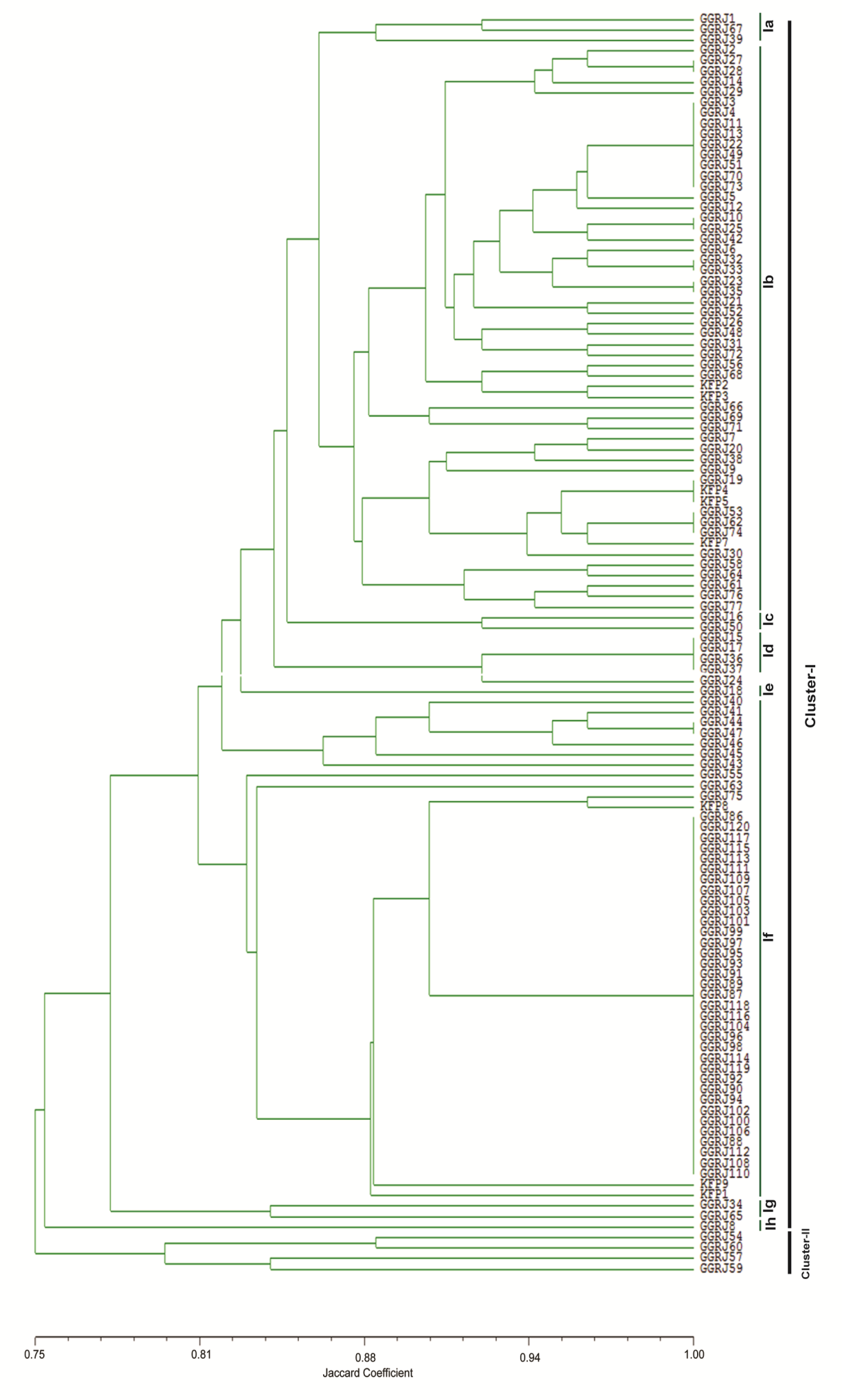
**

Supplement: Figure S2 — Dendrogram showing the genetic diversity of fluorescent pseudomonads of green gram rhizosphere. Clustering analysis based on the combined fingerprints of ERIC and BOX-PCR was performed using the UPGMA method followed by Jaccard’s coefficient. (DOCX) [file pone.0108378.s002.docx]

**Fig. S3**

**
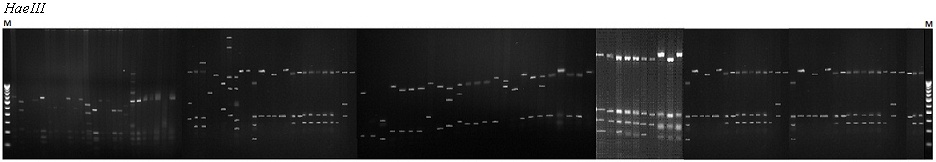
**


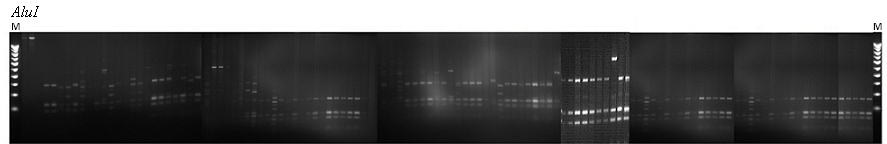


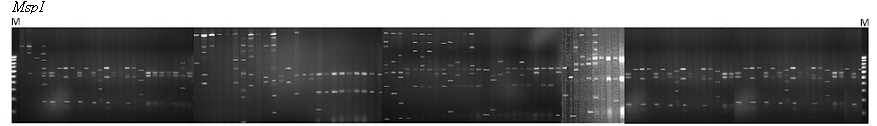


**(a)**

Supplement: Figure S3 — Restriction patterns of PCR amplified fragment of (a) 16S rDNA digested with HaeIII , AluI and MspI and (b) 16S–23S rDNA intergenic spacer region digestion with MspI . (DOCX) [file pone.0108378.s003.docx]

**Fig. S4**

**
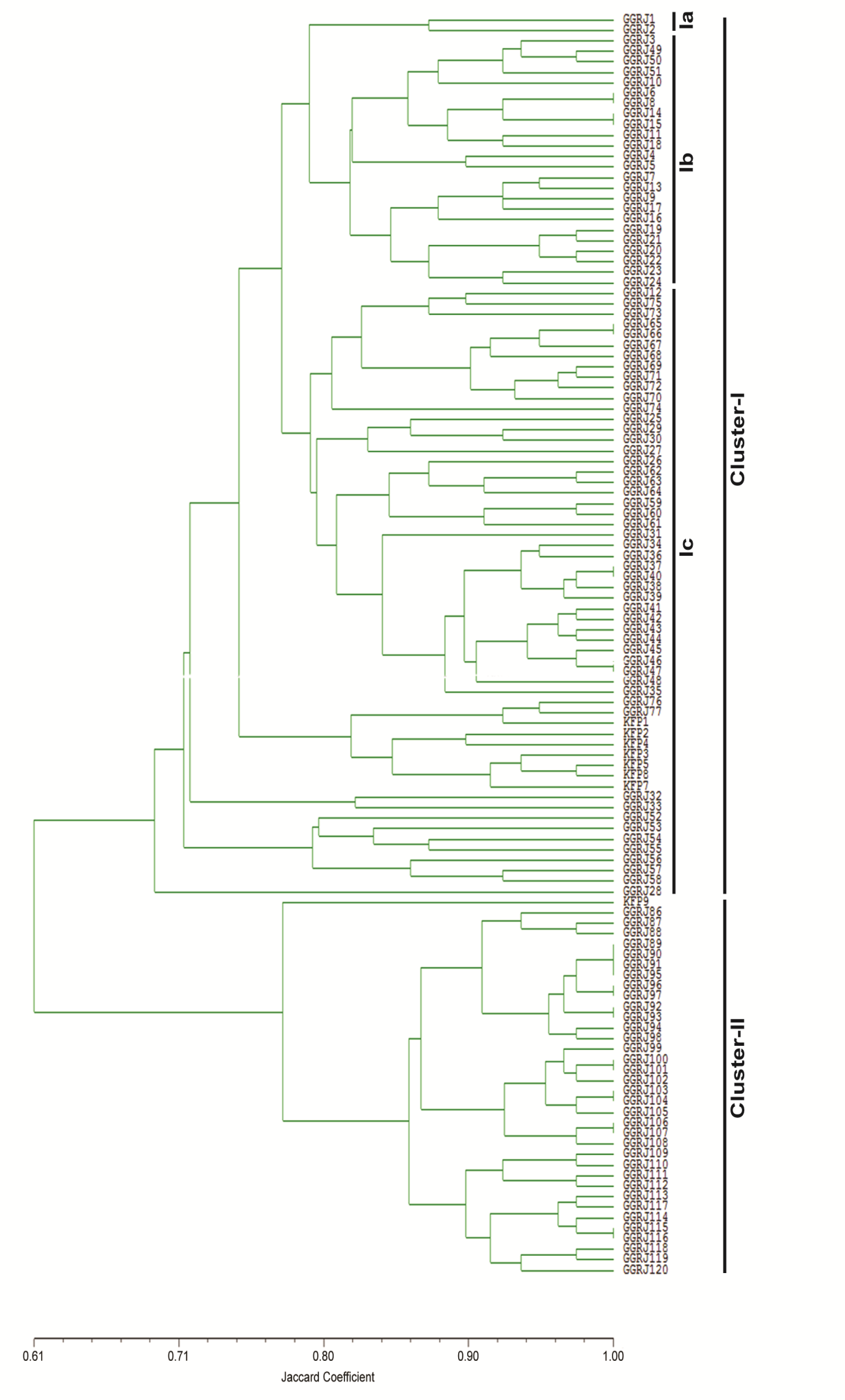
**

Supplement: Figure S4 — Dendrogram showing the genetic diversity of fluorescent pseudomonads of green gram rhizosphere. Clustering analysis of ARDRA fingerprints was performed using the UPGMA method followed by Jaccard’s coefficient. (DOCX) [file pone.0108378.s004.docx]

**
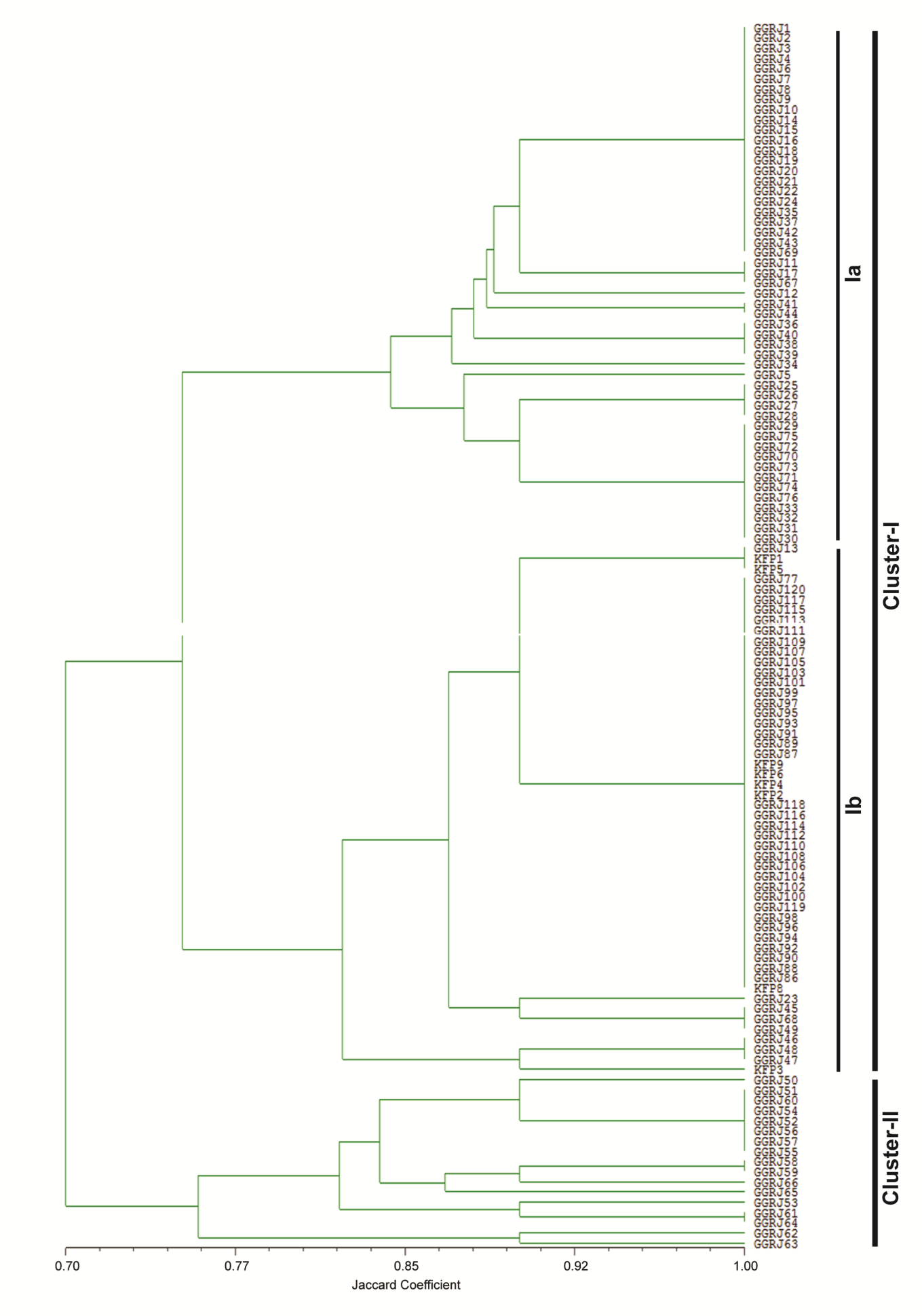
Fig. S5**

Supplement: Figure S5 — Dendrogram based on RISA analysis (restriction digestion of 16S–23S rDNA intergenic spacer region sequences by MspI ) showing the intra specific relationships among 120 members of the fluorescent pseudomonads. (DOCX) [file pone.0108378.s005.docx]

**Fig. S6**

**
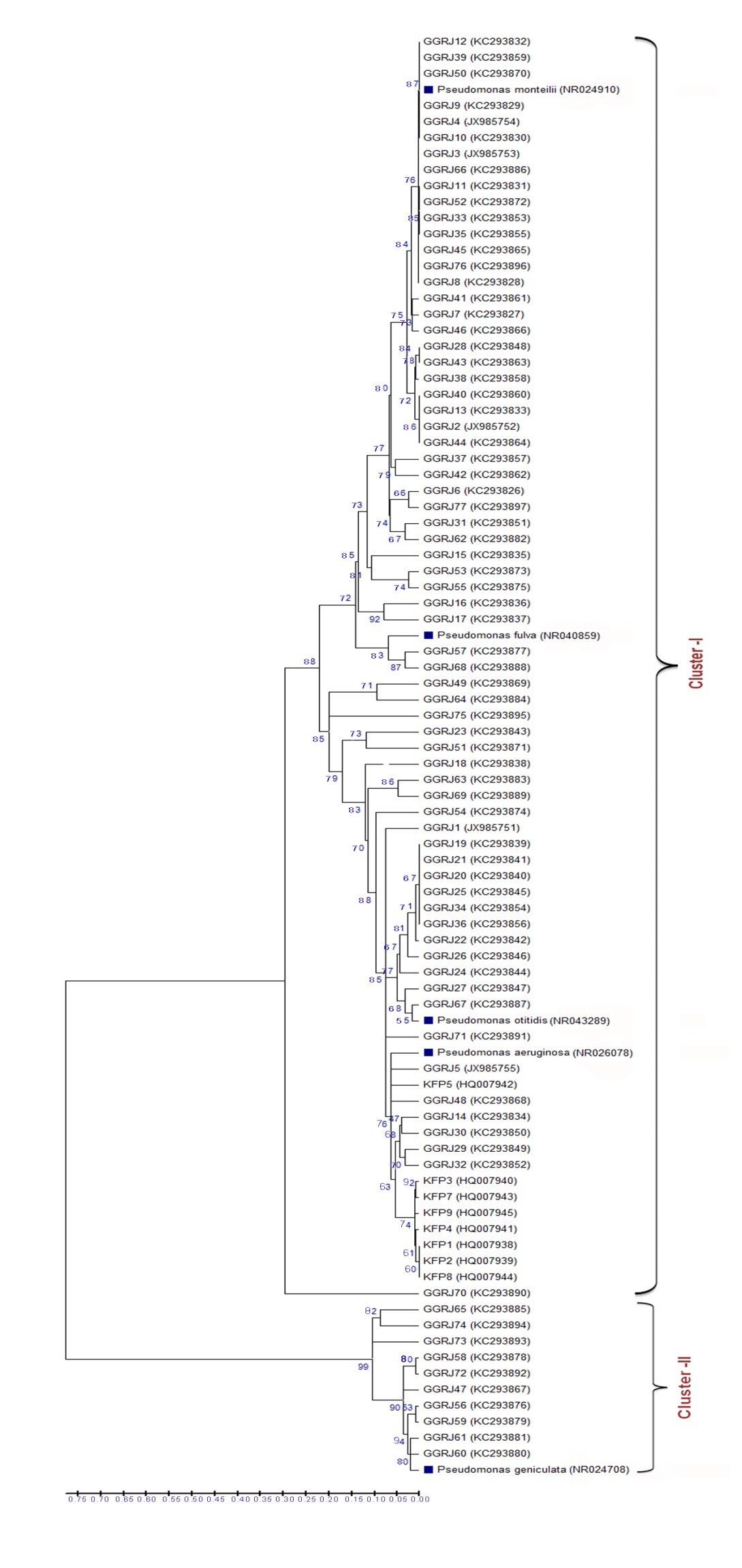
**

Supplement: Figure S6 — Phylogenetic analyses of fluorescent pseudomonads based on the nucleotide sequence of 16S rRNA using UPGMA method in MEGA 5.2. The Bar, 0.005, shows the substitutions per nucleotide position. (DOCX) [file pone.0108378.s006.docx]
