# Supplementary material for: Community Profiling of Culturable Fluorescent Pseudomonads in the Rhizosphere of Green Gram (Vigna radiata L.)
Source: PLoS One. 2014 Oct 3;9(10):e108378. doi: 10.1371/journal.pone.0108378 (PMC4184808; doi:10.1371/journal.pone.0108378)
Supplement: Figure S7 — Antifungal activity of purified phenazine-1-carboxylic acid (PCA) and pyoluteorin (PLT) against phytopathogenic fungi. (DOCX) [file pone.0108378.s007.docx]

**Fig. 7**

**
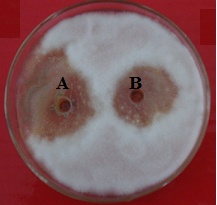
**

**Antifungal activity of purified PCA and PLT against (I) *Rhizoctonia solani and* (II) *F. oxysporum f. sp. ciceri***

1. **– purified PCA of GGRJ21**
2. **– purified PLT of GGRJ21**
3. **– purified PLT of GGRJ21**
4. **–purified PCA of GGRJ36**
5. **– purified PCA of GGRJ23**

**
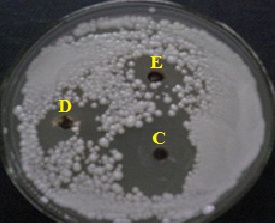
**
