# Supplementary material for: Community Profiling of Culturable Fluorescent Pseudomonads in the Rhizosphere of Green Gram (Vigna radiata L.)
Source: PLoS One. 2014 Oct 3;9(10):e108378. doi: 10.1371/journal.pone.0108378 (PMC4184808; doi:10.1371/journal.pone.0108378)
Supplement: Table S1 — Eighty-five Pseudomonas isolates with their taxonomic, phenotypic and biochemical characteristics. (DOCX) [file pone.0108378.s008.docx]

**Table S1**

| **Isolate code** | **REP PCR sub cluster type** | **ARDRA sub cluster type** | **RISA sub cluster type** | **Species level** | **NCBI accession no** | **Morphological and biochemical characteristic** | | | | | | | | | |
| --- | --- | --- | --- | --- | --- | --- | --- | --- | --- | --- | --- | --- | --- | --- | --- |
|  |  |  |  |  |  | **Gram Reaction** | **Shape** | **Motility** | **Fluorescent pigment** | **Catalase** | **Oxidase** | **Methyl red** | **Nitrate reduction** | **Strach hydrolysis** | **Gelatin liqification** |
| GGRJ1 | Ia | Ia | Ia | *Pseudomonas aeruginosa* | JX985751 | - | Rod | + | + | + | + | - | + | - | + |
| GGRJ2 | Ib | Ia | Ia | *Pseudomonas mosselii* | JX985752 | - | Rod | + | + | + | + | - | + | + | + |
| GGRJ3 | Ib | Ib | Ia | *Pseudomonas monteilii* | JX985753 | - | Rod | + | + | + | + | + | - | + | + |
| GGRJ4 | Ib | Ib | Ia | *Pseudomonas plecoglossicida* | JX985754 | - | Rod | + | + | + | + | + | + | + | + |
| GGRJ5 | Ib | Ib | Ia | *Pseudomonas aeruginosa* | JX985755 | - | Rod | + | + | + | + | - | - | - | + |
| GGRJ6 | Ib | Ib | Ia | *Pseudomonas monteilii* | KC293826 | - | Rod | + | + | + | + | - | + | + | + |
| GGRJ7 | Ib | Ib | Ia | *Pseudomonas plecoglossicida* | KC293827 | - | Rod | + | + | + | + | + | + | + | + |
| GGRJ8 | Ih | Ib | Ia | *Pseudomonas monteilii* | KC293828 | - | Rod | + | + | + | + | + | - | + | + |
| GGRJ9 | Ib | Ib | Ia | *Pseudomonas fulva* | KC293829 | - | Rod | + | + | + | + | + | + | + | + |
| GGRJ10 | Ib | Ib | Ia | *Pseudomonas taiwanensis* | KC293830 | - | Rod | + | + | + | + | + | + | + | + |
| GGRJ11 | Ib | Ib | Ia | *Pseudomonas taiwanensis* | KC293831 | - | Rod | + | + | + | + | + | - | + | + |
| GGRJ12 | Ib | Ic | Ia | *Pseudomonas fulva* | KC293832 | - | Rod | + | + | + | + | + | + | + | + |
| GGRJ13 | Ib | Ib | Ib | *Pseudomonas mosselii* | KC293833 | - | Rod | + | + | + | + | + | + | + | + |
| GGRJ14 | Ib | Ib | Ia | *Pseudomonas aeruginosa* | KC293834 | - | Rod | + | + | + | + | - | - | - | + |
| GGRJ15 | Id | Ib | Ia | *Pseudomonas chlororaphis* | KC293835 | - | Rod | + | + | + | + | - | + | + | + |
| GGRJ16 | Ic | Ib | Ia | *Pseudomonas brenneri* | KC293836 | - | Rod | + | + | + | + | - | + | + | + |
| GGRJ17 | Id | Ib | Ia | *Pseudomonas migulae* | KC293837 | - | Rod | + | + | + | + | - | - | + | + |
| GGRJ18 | Ie | Ib | Ia | *Pseudomonas otitidis* | KC293838 | - | Rod | + | + | + | + | - | + | - | + |
| GGRJ19 | Ib | Ib | Ia | *Pseudomonas aeruginosa* | KC293839 | - | Rod | + | + | + | + | - | + | - | + |
| GGRJ20 | Ib | Ib | Ia | *Pseudomonas otitidis* | KC293840 | - | Rod | + | + | + | + | - | - | - | + |
| GGRJ21 | Ib | Ib | Ia | *Pseudomonas aeruginosa* | KC293841 | - | Rod | + | + | + | + | - | + | - | + |
| GGRJ22 | Ib | Ib | Ia | *Pseudomonas otitidis* | KC293842 | - | Rod | + | + | + | + | - | - | - | + |
| GGRJ23 | Ib | Ib | Ib | *Pseudomonas nitroreducens* | KC293843 | - | Rod | + | + | + | + | + | - | + | + |
| GGRJ24 | Id | Ib | Ia | *Pseudomonas aeruginosa* | KC293844 | - | Rod | + | + | + | + | - | - | - | + |
| GGRJ25 | Ib | Ic | Ia | *Pseudomonas aeruginosa* | KC293845 | - | Rod | + | + | + | + | - | - | - | + |
| GGRJ26 | Ib | Ic | Ia | *Pseudomonas otidis* | KC293846 | - | Rod | + | + | + | + | - | - | - | + |
| GGRJ27 | Ib | Ic | Ia | *Pseudomonas aeruginosa* | KC293847 | - | Rod | + | + | + | + | + | + | - | + |
| GGRJ28 | Ib | Ic | Ia | *Pseudomonas mosselii* | KC293848 | - | Rod | + | + | + | + | + | + | + | + |
| GGRJ29 | Ib | Ic | Ia | *Pseudomonas resinovorans* | KC293849 | - | Rod | + | + | + | + | + | - | + | + |
| GGRJ30 | Ib | Ic | Ia | *Pseudomonas aeruginosa* | KC293850 | - | Rod | + | + | + | + | - | + | - | + |
| GGRJ31 | Ib | Ic | Ia | *Pseudomonas pseudoalcaligenes* | KC293851 | - | Rod | + | + | + | + | + | + | + | + |
| GGRJ32 | Ib | Ic | Ia | *Pseudomonas aeruginosa* | KC293852 | - | Rod | + | + | + | + | - | - | - | + |
| GGRJ33 | Ib | Ic | Ia | *Pseudomonas entomophila* | KC293853 | - | Rod | + | + | + | + | - | + | + | + |
| GGRJ34 | Ig | Ic | Ia | *Pseudomonas otidis* | KC293854 | - | Rod | + | + | + | + | - | - | - | + |
| GGRJ35 | Ib | Ic | Ia | *Pseudomonas fulva* | KC293855 | - | Rod | + | + | + | + | + | + | + | + |
| GGRJ36 | Id | Ic | Ia | *Pseudomonas aeruginosa* | KC293856 | - | Rod | + | + | + | + | - | + | - | + |
| GGRJ37 | Id | Ic | Ia | *Pseudomonas fulva* | KC293857 | - | Rod | + | + | + | + | + | - | + | + |
| GGRJ38 | Ib | Ic | Ia | *Pseudomonas mosselii* | KC293858 | - | Rod | + | + | + | + | + | + | + | + |
| GGRJ39 | Ia | Ic | Ia | *Pseudomonas flavescens* | KC293859 | - | Rod | + | + | + | + | + | - | + | + |
| GGRJ40 | If | Ic | Ia | *Pseudomonas mosselii* | KC293860 | - | Rod | + | + | + | + | + | + | + | + |
| GGRJ41 | If | Ic | Ia | *Pseudomonas entomophila* | KC293861 | - | Rod | + | + | + | + | + | + | + | + |
| GGRJ42 | Ib | Ic | Ia | *Pseudomonas oryzihabitans* | KC293862 | - | Rod | + | + | + | + | + | - | + | + |
| GGRJ43 | If | Ic | Ia | *Pseudomonas oryzihabitans* | KC293863 | - | Rod | + | + | + | + | + | + | + | + |
| GGRJ44 | If | Ic | Ia | *Pseudomonas umsongensis* | KC293864 | - | Rod | + | + | + | + | + | + | + | + |
| GGRJ45 | If | Ic | Ib | *Pseudomonas jessenii* | KC293865 | - | Rod | + | + | + | + | + | - | + | + |
| GGRJ46 | If | Ic | Ib | *Pseudomonas monteilii* | KC293866 | - | Rod | + | + | + | + | + | + | + | + |
| GGRJ47 | If | Ic | Ib | *Pseudomonas hibiscicola* | KC293867 | - | Rod | + | + | + | + | + | + | + | + |
| GGRJ48 | Ib | Ic | Ib | *Pseudomonas otitidis* | KC293868 | - | Rod | + | + | + | + | + | - | - | + |
| GGRJ49 | Ib | Ib | Ib | *Pseudomonas plecoglossicida* | KC293869 | - | Rod | + | + | + | + | + | + | + | + |
| GGRJ50 | Ic | Ib | II | *Pseudomonas monteilii* | KC293870 | - | Rod | + | + | + | + | + | + | + | + |
| GGRJ51 | Ib | Ib | II | *Pseudomonas nitroreducens* | KC293871 | - | Rod | + | + | + | + | + | - | + | + |
| GGRJ52 | Ib | Ic | II | *Pseudomonas plecoglossicida* | KC293872 | - | Rod | + | + | + | + | + | + | + | + |
| GGRJ53 | Ib | Ic | II | *Pseudomonas plecoglossicida* | KC293873 | - | Rod | + | + | + | + | + | + | + | + |
| GGRJ54 | II | Ic | II | *Pseudomonas otitidis* | KC293874 | - | Rod | + | + | + | + | + | + | - | + |
| GGRJ55 | If | Ic | II | *Pseudomonas monteilii* | KC293875 | - | Rod | + | + | + | + | + | + | + | + |
| GGRJ56 | Ib | Ic | II | *Pseudomonas geniculata* | KC293876 | - | Rod | + | + | + | + | + | + | + | + |
| GGRJ57 | II | Ic | II | *Pseudomonas argentinensis* | KC293877 | - | Rod | + | + | + | + | + | + | + | + |
| GGRJ58 | Ib | Ic | II | *Pseudomonas geniculata* | KC293878 | - | Rod | + | + | + | + | + | + | + | + |
| GGRJ59 | II | Ic | II | *Pseudomonas geniculata* | KC293879 | - | Rod | + | + | + | + | + | - | + | + |
| GGRJ60 | II | Ic | II | *Pseudomonas hibiscicola* | KC293880 | - | Rod | + | + | + | + | + | - | + | + |
| GGRJ61 | Ib | Ic | II | *Pseudomonas geniculata* | KC293881 | - | Rod | + | + | + | + | + | + | + | + |
| GGRJ62 | Ib | Ic | II | *Pseudomonas nitroreducens* | KC293882 | - | Rod | + | + | + | + | + | - | + | + |
| GGRJ63 | If | Ic | II | *Pseudomonas otitidis* | KC293883 | - | Rod | + | + | + | + | + | - | - | + |
| GGRJ64 | Ib | Ic | II | *Pseudomonas otitidis* | KC293884 | - | Rod | + | + | + | + | + | - | - | + |
| GGRJ65 | Ig | Ic | II | *Pseudomonas boreopolis* | KC293885 | - | Rod | + | + | + | + | + | - | + | + |
| GGRJ66 | Ib | Ic | II | *Pseudomonas plecoglossicida* | KC293886 | - | Rod | + | + | + | + | + | + | + | + |
| GGRJ67 | Ia | Ic | Ia | *Pseudomonas nitroreducens* | KC293887 | - | Rod | + | + | + | + | + | - | + | + |
| GGRJ68 | Ib | Ic | Ib | *Pseudomonas pseudoalcaligenes* | KC293888 | - | Rod | + | + | + | + | + | - | + | + |
| GGRJ69 | Ib | Ic | Ia | *Pseudomonas otitidis* | KC293889 | - | Rod | + | + | + | + | + | - | - | + |
| GGRJ70 | Ib | Ic | Ia | *Pseudomonas pohangensis* | KC293890 | - | Rod | + | + | + | + | + | + | + | + |
| GGRJ71 | Ib | Ic | Ia | *Pseudomonas otitidis* | KC293891 | - | Rod | + | + | + | + | + | - | - | + |
| GGRJ72 | Ib | Ic | Ia | *Pseudomonas geniculata* | KC293892 | - | Rod | + | + | + | + | + | - | + | + |
| GGRJ73 | Ib | Ic | Ia | *Pseudomonas boreopolis* | KC293893 | - | Rod | + | + | + | + | + | - | + | + |
| GGRJ74 | Ib | Ic | Ia | *Pseudomonas geniculata* | KC293894 | - | Rod | + | + | + | + | + | - | + | + |
| GGRJ75 | If | Ic | Ia | *Pseudomonas otitidis* | KC293895 | - | Rod | + | + | + | + | + | - | - | + |
| GGRJ76 | Ib | Ic | Ia | *Pseudomonas plecoglossicida* | KC293896 | - | Rod | + | + | + | + | + | + | + | + |
| GGRJ77 | Ib | Ic | Ib | *Pseudomonas pseudoalcaligenes* | KC293897 | - | Rod | + | + | + | + | + | + | + | + |
| KFP1 | If | Ic | Ib | *Pseudomonas aeruginosa* | HQ007938 | - | Rod | + | + | + | + | - | + | - | + |
| KFP2 | Ib | Ic | Ib | *Pseudomonas aeruginosa* | HQ007939 | - | Rod | + | + | + | + | - | + | - | + |
| KFP3 | Ib | Ic | Ib | *Pseudomonas aeruginosa* | HQ007940 | - | Rod | + | + | + | + | - | + | - | + |
| KFP4 | Ib | Ic | Ib | *Pseudomonas aeruginosa* | HQ007941 | - | Rod | + | + | + | + | - | + | - | + |
| KFP5 | Ib | Ic | Ib | *Pseudomonas aeruginosa* | HQ007942 | - | Rod | + | + | + | + | - | + | - | + |
| KFP7 | Ib | Ic | Ib | *Pseudomonas aeruginosa* | HQ007943 | - | Rod | + | + | + | + | - | + | - | + |
| KFP8 | If | Ic | Ib | *Pseudomonas aeruginosa* | HQ007944 | - | Rod | + | + | + | + | - | + | - | + |
| KFP9 | If | II | Ib | *Pseudomonas aeruginosa* | HQ007945 | - | Rod | + | + | + | + | - | - | - | + |

+ means positive for the test, - means negative for the test
