# Supplementary material for: Community Profiling of Culturable Fluorescent Pseudomonads in the Rhizosphere of Green Gram (Vigna radiata L.)
Source: PLoS One. 2014 Oct 3;9(10):e108378. doi: 10.1371/journal.pone.0108378 (PMC4184808; doi:10.1371/journal.pone.0108378)
Supplement: Table S2 — Quantitative estimation of indoles with tryptophan concentrations of 100, 200 and 500 µg ml−1. Values are mean of three replicates. (DOCX) [file pone.0108378.s009.docx]

**Table S2**

| **Fluorescent pseudomonads** | **Indole production (µg ml^-1^)**  **at 0 µg ml^-1^ tryptophan** | **Indole production (µg ml^-1^)**  **at 50µg ml^-1^ tryptophan** | **Indole production (µg ml^-1^)**  **at 200µg ml^-1^ tryptophan** | **Indole production (µg ml^-1^)**  **at 500µg ml^-1^ tryptophan** |
| --- | --- | --- | --- | --- |
| GGRJ1 | 1.25±0.65 | 93.03±1 | 30.64±0.58 | 23.17±1.77 |
| GGRJ5 | 2.46±0.32 | 119.76±0.85 | 41±1.48 | 33.18±1.01 |
| GGRJ7 | 0 | 66.36±1.69 | 23.44±1.64 | 17.8±0.77 |
| GGRJ9 | 0 | 56.1±0.49 | 20.17±0.9 | 13.35±0.78 |
| GGRJ12 | 0 | 100.55±1.47 | 34.15±1.1 | 27.5±0.56 |
| GGRJ14 | 1.11±0.24 | 154.47±4.27 | 86.84±1.67 | 53.87±2.14 |
| GGRJ15 | 0 | 44.11±0.95 | 12.87±1.53 | 6.62±0.56 |
| GGRJ18 | 0 | 49.15±0.89 | 13.8±1.63 | 8.54±0.79 |
| GGRJ19 | 1.34±0.12 | 175.1±1.95 | 121.16±1.85 | 67.87±0.6 |
| GGRJ21 | 2.84±0.67 | 186.13±2.68 | 143.36±1.48 | 71.35±1 |
| GGRJ23 | 0 | 42.46±0.72 | 11.22±0.94 | 4.31±0.84 |
| GGRJ24 | 1.45±0.84 | 132.33±2.08 | 45.18±1.15 | 33.57±1.02 |
| GGRJ25 | 0 | 148.21±3.52 | 66.87±1.39 | 48.24±1.08 |
| GGRJ27 | 0 | 160.51±1.48 | 95.63±1.59 | 61.17±0.84 |
| GGRJ29 | 0 | 175.13±2.01 | 132.4±1.63 | 69.1±0.58 |
| GGRJ31 | 2±0.98 | 88.47±1.13 | 29.45±0.59 | 21.28±0.75 |
| GGRJ33 | 0 | 67.75±0.47 | 27.24±1.1 | 18.5±0.71 |
| GGRJ35 | 0 | 48.36±0.84 | 16.09±1.66 | 10.42±0.89 |
| GGRJ36 | 1.34±0.21 | 56.94±1.75 | 23.13±1.87 | 16.42±0.88 |
| GGRJ39 | 0 | 109.2±0.85 | 36.55±1.17 | 30.13±0.9 |
| GGRJ42 | 0 | 134.83±2.49 | 55.17±0.96 | 45.51±0.64 |
| GGRJ43 | 2.14±0.43 | 122.46±1.65 | 42.1±1.05 | 35.76±0.4 |
| GGRJ51 | 0 | 147.6±1.35 | 73.6±1.51 | 52.95±0.96 |
| KFP1 | 0 | 164.76±1.07 | 107.46±2.33 | 65.1±0.89 |
| KFP2 | 1±0.41 | 133.53±1.42 | 49.25±0.9 | 41.5±1.68 |
