# Supplementary material for: Community Profiling of Culturable Fluorescent Pseudomonads in the Rhizosphere of Green Gram (Vigna radiata L.)
Source: PLoS One. 2014 Oct 3;9(10):e108378. doi: 10.1371/journal.pone.0108378 (PMC4184808; doi:10.1371/journal.pone.0108378)
Supplement: Table S3 — Optical density (OD) of 10 Pseudomonas isolates at 600 nm after 24 hours of growth under different osmotic stress condition considering normal growth in nutrient broth (NB) as control. Values are mean of three replicates. (DOCX) [file pone.0108378.s010.docx]

**Table S3**

| **Isolate code** | **Control** | **-0.05MPa** | **-0.15MPa** | **-0.3 MPa** | **-0.49MPa** | **-0.73MPa** |
| --- | --- | --- | --- | --- | --- | --- |
| **GGRJ5** | **2.91±0.59** | **1.44±0.11** | **1.12±0.15** | **0.79±0.07** | **0.51±0.12** | **0.21±0.23** |
| **GGRJ9** | **2.72±0.73** | **1.41±0.04** | **1.23±0.21** | **0.62±0.01** | **0.54±0.02** | **0.13±0.13** |
| **GGRJ13** | **2.94±0.67** | **1.52±0.02** | **1.31±0.03** | **0.71±0.02** | **0.43±0.03** | **0.14±0.02** |
| **GGRJ17** | **2.90±0.32** | **1.47±0.12** | **1.39±0.14** | **0.67±0.12** | **0.48±0.04** | **0.11±0.21** |
| **GGRJ18** | **2.96±0.88** | **1.52±0.13** | **1.42±0.05** | **0.45±0.41** | **0.28±0.02** | **0.12±0.03** |
| **GGRJ21** | **2.83±0.52** | **2.79±0.02** | **2.75±0.01** | **2.67±0.03** | **2.36±0.12** | **1.67±0.04** |
| **GGRJ25** | **2.87±0.83** | **1.54±0.12** | **1.46±0.23** | **0.56±0.11** | **0.31±0.12** | **0.22±0.01** |
| **GGRJ34** | **2.92±0.53** | **1.47±0.23** | **1.41±0.13** | **0.64±0.22** | **0.35±0.02** | **0.24±0.04** |
| **GGRJ36** | **2.88±0.71** | **1.56±0.03** | **1.42±0.03** | **0.53±0.31** | **0.41±0.04** | **0.14±0.07** |
| **GGRJ39** | **2.85±0.43** | **1.54±0.07** | **1.47±0.21** | **0.64±0.14** | **0.34±0.13** | **0.11±0.03** |
